# Identifying omic biomarkers for chronic inflammatory diseases associated with periodontitis using percolation on multi-disease gene co-expression networks

**DOI:** 10.1038/s43856-026-01591-w

**Published:** 2026-04-21

**Authors:** Xingyu Wang, Lei Liu, Fan Yang, Chuling Huang, Ziyi Mei, Jie Li, Shiyong Ma

**Affiliations:** 1 Basic Medicine Research and Innovation Center for Novel Target and Therapeutic Intervention, The Ministry of Education, College of Pharmacy, Chongqing Medical University, Chongqing, China; 2 Medical Center of Hematology, State Key Laboratory of Trauma and Chemical Poisoning, Chongqing Key Laboratory of Hematology and Microenvironment, Xinqiao Hospital of Army Medical University, Chongqing, China; 3 College of Stomatology, Chongqing Key Laboratory of Oral Diseases and Biomedical Sciences, Chongqing Municipal Key Laboratory of Oral Biomedical Engineering of Higher Education, Chongqing Medical University, Chongqing, China

**Keywords:** Computational biology and bioinformatics, Systems biology

## Abstract

**Background:**

Chronic inflammatory diseases, such as ulcerative colitis (UC), Crohn’s disease (CD), Alzheimer’s disease (AD) and Parkinson’s disease (PD) are clinically related to periodontitis. However, the computation of omic biomarkers regarding these diseases has not leveraged this association.

**Methods:**

We developed PMGCN, a computational framework that employs optimal percolations on multi-disease gene co-expression networks derived from bulk transcriptomic gene expression profiles to identify a parsimonious set of key nodes as candidate omic biomarkers.

**Results:**

Evaluation of PMGCN on independent clinical studies of four chronic inflammatory diseases demonstrates improved predictive performance evaluated via cross validation with bootstrapping compared to commonly used univariate differentially expressed genes. Specifically for UC, three key gene biomarkers (CXCL5, FOSB, PTGR1) are identified by PMGCN, and public single-cell RNA-seq datasets confirm that the mainly altered inflammation signaling pathways in three cell clusters are connected to UC and periodontitis progression.

**Conclusions:**

PMGCN proposes a computational biomarker identification approach leveraging multi-disease association, the discovered gene biomarkers demonstrate improved prediction of chronic inflammatory diseases and provide novel insights into disease progression.

## Abstract (web summary)

Wang, Liu et al. develop PMGCN, a computational method that apply optimal percolation to multi-disease gene networks for biomarker discovery. It improves prediction for several chronic inflammatory diseases and uncovers shared inflammation pathways.

## Plain language summary

Many chronic inflammatory diseases, like certain bowel and brain conditions, are known to be linked to the gum disease periodontitis. This study aimed to find key genes involved in these connections to help predict disease risk. We created a computer tool called PMGCN, to analyze gene activities across these related diseases utilizing optimal percolation (targeted knockout of key connectors). This approach identifies a small set of the most important genes, which were better at predicting several chronic inflammatory diseases. For ulcerative colitis, three specific genes were highlighted, and further analyses showed they are active in immune cells relevant to both gum and gut disease. This research offers a new way to find biomarkers for multiple linked diseases, which could lead to improved disease detection, and a better understanding of how these conditions develop together.

## Introduction

Chronic inflammatory diseases have been recognized as the greatest threat of health in the current world, with more than 50% of all deaths being attributable to inflammation-related diseases^1^. Interestingly, periodontitis as an oral inflammatory disease not only affects oral health^2^, but is associated with several chronic inflammatory diseases such as ulcerative colitis (UC), Crohn’s disease (CD) and Alzheimer’s disease (AD)^3–6^, as highlighted by multiple observational studies. Recent studies suggested that the biological mechanisms linking periodontitis with other inflammatory diseases include microbial dysbiosis, immune cell activation and cytokine production^7–12^. Despite the epidemiological and mechanistic correlations between chronic inflammatory diseases and periodontitis, computational methods of biomarker discovery for chronic inflammatory diseases have not fully leveraged this association.

Recent studies have utilized bioinformatics methods to identify crosstalk signaling pathways and genes associated with periodontitis and other diseases mainly by analyzing the common differentially expressed genes^13–17^. Notably, these analyses prioritized individual genes mainly using the node degree in protein-protein interaction network or relying on the overlaps of WGCNA^18^ modules. More generally, network biology approaches for multi-disease analysis primarily rely on network comparing methods for identifying disease-specific and shared biomarkers, potentially suggesting new therapeutic targets^19^. These approaches often starts with inferring condition-specific co-expression or correlation networks from omics data, and then proceeds through multi-scale comparisons: evaluating global topological properties (e.g., network density)^20–22^; assessing local node-level metrics (e.g., degree and betweenness centrality)^23,24^; identifying differential edges via statistical testing^25^, network alignment^26^, graphlet-based analysis^27^, etc.; and module-level comparative analyses^28,29^. However, while these current approaches excel at mapping similarities and differences in genes, edges, or modules between network architectures, they may not directly pinpoint the most critically vulnerable elements whose targeted disruption would maximally impair the disease-associated network state - a potential key consideration for therapeutic intervention and biomarker discovery. To address this, we introduce a network dismantling strategy into the multi-disease analysis paradigm.

Percolation, a physics concept akin to the permeation of a liquid through a porous membrane, impairs network connectivity by deactivating links or nodes. During the percolation attack process, the size of the network’s largest mutually connected component (LMCC) decreases sharply as network impairment increases to a certain limit, reflecting a phase transition. Percolation has been applied to analyze the transmission patterns in epidemic spreading^30,31^ and brain connectome^32,33^. Optimal percolation methods aim to iteratively identify a parsimonious set of important nodes that are able to dismantle the network connectivity, which has been studied in both single-layer networks and more recently in multilayer networks^34,35^. Compared with single-layer networks, multilayer networks can capture more complex and heterogeneous interactions by explicitly modeling multiple types of relationships or conditions within a unified framework^36^. Among them, multiplex networks represent a specific class of multilayer networks in which each node appears in multiple layers and is connected only to its counterparts across layers^37^. Multiplex networks have been adopted to successfully represent various real-world complex systems, including social networks^38^ technology systems^39^ and biological systems^40–42^.

Together, a comprehensive evaluation of gene importance for multi-disease via the integrative multilayer gene correlation network is essential, and advances in integrative analyzing multiplex networks using optimal percolation make it possible to identify minimum core nodes as key omic biomarkers from a multi-disease perspective.

In this context, we present Percolation on Multi-disease Gene Co-expression Network (PMGCN) approach. We constructed multi-disease gene co-expression networks as multiplex networks. Each network consisted of one layer for periodontitis and another for a different chronic inflammatory disease. We then applied optimal percolation algorithms to each multiplex network to identify a minimal omics biomarker set. We specifically selected Periodontitis-UC as a primary, in-depth case to validate the biological plausibility of the PMGCN-derived biomarkers, while the general applicability of the framework was confirmed using other disease pairs. For the Periodontitis-UC network, CXCL5, FOSB and PTGR1 were identified as the key biomarker set, the functional annotation analysis combined with investigations of large amounts of scRNA-Seq data reveals that altered inflammation signaling pathway in different cell clusters including fibroblasts, epithelial cells and B cells are connected to UC and periodontitis progression. Finally, our findings indicated that the gene biomarkers screened by PMGCN exhibited higher accuracy in predicting several chronic inflammatory diseases apart from UC. Taken together, this work introduces PMGCN, a computational framework for identifying parsimonious and robust omic biomarker sets for chronic inflammatory diseases by leveraging the multi-disease associations, highlighting its significance in explaining the clinical correlation of multi-disease and enhancing disease prediction.

## Methods

### Data pre-processing and gene co-expression network computation

#### Setting up bulk gene-expression dataset

We obtained 8 bulk microarray datasets for periodontitis and four other chronic inflammatory diseases: Ulcerative Colitis (UC), Crohn’s Disease (CD), Alzheimer’s Disease, and Parkinson’s Disease (Supplementary Table 1). For diseases with multiple datasets (UC and CD), we selected one dataset each (UC1 and CD1) for PMGCN network construction and biomarker development, reserving all others for independent testing.

#### Data pre-processing and normalization

All the microarray datasets used in the study for gene co-expression network construction were pre-processed using a uniform pipeline. For the obtained microarray expression data, the microarray probe ID for the datasets were first converted to gene symbol based on the annotation information of the microarray platform respectively. Gene symbols were then compared with the gene list in HGNC database (https://www.genenames.org/download/custom/), and gene symbols that do not match were removed for further analysis. To deal with the duplicated gene symbols, average expression levels for each gene symbol were calculated and the one with the maximum expression was kept. The quantiles (0–100%) were calculated to determine whether the expression values need to be log transformed (base = 2).

#### Differential expression genes and functional analysis

Differential expression analysis was performed on the preprocessed data matrix for each dataset using *limma (v3.54.2)*, and differentially expressed genes (DEGs) between disease samples and the corresponding controls (adj.P < 0.05 & |log_2_FC | > 1) were obtained for each disease respectively. The common DEGs, which are defined as the genes that are consistently dysregulated in both diseases, were obtained by finding the intersection of periodontitis-DEGs and DEGs of one chronic inflammatory disease. Volcano plots were created using *ggplot2 (v3.4.2)*, and the heatmaps were generated using *pheatmap (v1.0.12)*. Gene ontology (GO) analysis and Kyoto Encyclopedia of Genes and Genomes (KEGG) pathway enrichment analysis were conducted using *clusterProfiler (v4.6.2)*. Benjamini-Hochberg (*BH*) method was used for the multiple testing correction of the *p* values (adj.*P* < 0.05).

#### Construction of the multi-disease gene co-expression networks

To ensure that a plausible number of nodes and edges are contained in the constructed gene co-expression networks, a tailored network construction process utilizing an expanded pool of differential expressed genes was developed. First, differentially expressed genes with a less stringent cutoff (adj.*P* < 0.05) were obtained for the Periodontitis and one of the investigated chronic inflammatory disease datasets respectively; second, for each disease within one disease pair, $\rho$ percent of the top $\left|{\log }_{2}{FC}\right|$ genes with significant adj.*P* values were obtained, and the parameter $\rho$ was determined through grid searching in order to ensure a plausible size of the network; the intersecting genes (referred to as common DEGs) for each disease pair (periodontitis and one of the investigated chronic inflammatory disease) was applied for the corresponding construction of gene co-expression networks. *GeneNet (v1.2.16)* was utilized to estimate the correlation coefficient between genes based on the expression data using the dynamic partial correlation method. The Gaussian graphical model was subsequently used to estimate the probability of network edges, and edges with probabilities greater than $\alpha$ were extracted to form the adjacency matrix of the unweighted and undirected network, the threshold $\alpha$ was tuned jointly with parameter $\rho$ through grid searching. Multi-disease networks for each disease pair were constructed in the form of multiplex networks with one layer representing periodontitis and one layer representing one of the investigated chronic inflammatory diseases (Supplementary Fig. 1).

### Optimal percolations on multi-disease networks

Our proposed PMGCN algorithm adopted an integrated computational framework incorporating several heuristic optimal percolation algorithms to improve the network fragmentation efficiency and reduce the size of node sequences for biomarker discovery.

#### Preliminary

A Multi-Disease Network ${A}^{m}$ of $m$ layers in the form of an unweighted and undirected multiplex network is denoted as:

1 $${A}^{m}=\{{A}^{\left[1\right]},{A}^{[2]},\ldots ,{A}^{[m]}\,\},$$

where ${A}^{[m]}$ denotes the adjacency matrix for layer $m$, ${A}_{{ij}}^{[m]}=1$ if and only if node $i$ and $j$ are the endpoints of an edge at layer $m$. We denote by ${V}^{m}$ the node set and by ${E}^{m}$ the edge set for layer $m$. Without loss of generality, the multiplex networks are assumed to be composed of the same set of nodes for different layers. i.e.

2 $${V}^{[1]}={V}^{[2]}=\cdots ={V}^{\left[m\right]}=V,\left|V\right|=n,$$

where $\left|V\right|$ represents the cardinality of node set $V$. For the multiplex network ${A}^{m}$, interlayer links among layers are created, by which nodes in one layer are connected to the counterparts of other layers.

Particularly, for the Multi-Disease Networks in this paper which contains one Periodontitis layer and one chronic inflammatory disease layer, a two-layer multiplex network ${A}^{2}$ which is composed of layers ${A}^{[1]}$ and ${A}^{[2]}$ was used. For both of the two layers, the node set is composed of the common DEGs of the disease pair, which means ${V}^{[1]}={V}^{[2]}$. However, the ${E}^{[1]}$ and ${E}^{[2]}$ present different topologies and structures.

Our main objective is to determine a minimum and plausible gene sequence $S$ for such Multi-Disease Networks, such that by iteratively removing the gene in $S$ and propagating the loss of edges across layers, the network can be decomposed into non-extended connected components. We assume in this article that these parsimonious sets of genes for the Multi-Disease Networks are key nodes that are important for both the two diseases, and to identify omic biomarkers for chronic inflammatory diseases leveraging gene associations of the associated disease periodontitis is able to contribute to improving omic biomarker discovery.

To dismantle the created Multi-Disease Networks by a minimum size of such sequence $S$, an intrinsically difficult combinatorial optimization issue exists for the optimal percolation problem, and the computational complexity of which belongs to the NP-hard (non-deterministic polynomial hard) class. Therefore, so far, the optimal percolation approaches have mainly been relying on heuristic methods for node ordering and target selection. In this article, in an attempt to cut down the number of biomarkers selected, given that no consensus has been reached on the best percolation approaches, several heuristic methods have been applied, and these approaches could be categorized into three types of strategies which are listed below.

#### Strategies relying on network cores

Heuristic strategies of this category are based on network cores. K-core is defined as a subgraph in which all vertices have at least degree $K$. K-core^43^ ranking method ranks the node by the core number of a node for each layer, the core number of a node is defined as the largest value $K$ such that the node is part of a k-core of the layer. CoreHD^44^ method extract the 2-core of the network and then ranks the node by its degree for each layer. Both methods are single-layer ranking methods.

#### Strategies relying on node centrality

The second type of strategy relies on a certain form of node centrality. Highest Degree Adaptive (HDA)^34^ method computes the product of the degrees of each layer for one node, and rank that node by the product value. Effective Multiplex Degree (EMD)^45^ assigns a weight ${\omega }_{i}$ to each node $i$ according to the equation below:

3 $${\omega }_{i}={\sum }_{m=1}^{2}{\sum }_{j\epsilon {N}_{i}^{(m)}}\frac{1}{{\Gamma }_{j}}\frac{{\omega }_{j}}{{q}_{j}^{(m)}}$$

where ${N}_{i}^{(m)}$ represents the neighbors for node $i$ in the layer $m$, ${\Gamma }_{j}$ represents the number of layers in which node $j$ exits, and ${q}_{j}^{(m)}$ is the degree of node $j$ in the layer $m$. EMD1step^45^ is a simplified version of EMD algorithm, the equation is given below:

4 $${\widetilde{\omega }}_{i}={\sum }_{m=1}^{2}{\sum }_{j\epsilon {N}_{i}^{(m)}}\frac{1}{{\Gamma }_{j}}\frac{{Q}_{j}}{{q}_{j}^{(m)}}$$

where $${Q}_{j}$$ denotes:

5 $${Q}_{j}={\sum }_{m=1}^{2}{q}_{j}^{(m)}$$

The difference between the two algorithms is that EMD1step replaces the weight ${\omega }_{j}$ with sum of degree ${Q}_{j}$ for the node $j$ to get rid of the computational burden induced by the self-referential calculations. In brief, EMD/EMD1step algorithms are able to take the heterogeneities between layer degrees and within the neighborhood of a node into account to measure the effect of the removal of the node.

#### Strategies relying on collective influence

This type of strategy measures the importance of nodes by the defined Collective Influence (CI)^46^ to reduce computation burden. CI algorithm assigns a ${{CI}}_{l}\left(i\right)$ score to node $i$ for each layer, and the equation of CI algorithm is given below:

6 $${{CI}}_{l}\left(i\right)=({q}_{i}-1){\sum }_{j\epsilon \partial B(i,l)}({q}_{j}-1)$$

where $\partial B(i,l)$ denotes the boundary of the ball of radius $l$ around node $i$. CI2 is an implementation of CI algorithm with $l=2$. The nodes with higher CI scores tend to be responsible for the regulations of larger number of communications between nodes.

DCI (Duplex Collective Influence) and DCIz algorithms^35^ generalized CI algorithm to duplex networks. DCI was defined as follows:

7 $${DCI}\left(i\right)=\frac{{q}_{i}^{[1]}{q}_{i}^{[2]}-{q}_{i}^{{int}}}{{q}_{i}^{{aggr}}}[{\sum }_{j}{A}_{{ij}}^{\left[1\right]}({k}_{j}^{[2]}-1)+{A}_{{ij}}^{\left[2\right]}({k}_{j}^{[1]}-1)]$$

where ${q}_{i}^{{int}}$ denotes the degree of node $i$ in the intersection graph (i.e., the graph containing only the links which appear in both layers) for the duplex networks, and ${q}_{i}^{{aggr}}$ denotes the degree of node $i$ in the binary aggregated graph which is defined as the union graph contained the connections of both the two layers. DCIz is a modified version of DCI score, and the equation is as follows:

8 $${DCIz}\left(i\right)=	 \frac{\left({q}_{i}^{\left[1\right]}+1\right)\left({q}_{i}^{\left[2\right]}+1\right)-{3q}_{i}^{{int}}-1}{{q}_{i}^{{aggr}}}\\ 	\left[{\sum }_{j}{A}_{{ij}}^{\left[1\right]}({k}_{j}^{[2]}-1) + {A}_{{ij}}^{\left[2\right]}({k}_{j}^{[1]}-1)\right]$$

#### Pareto optimization

Briefly, Pareto optimality or Pareto efficiency^47^ originally indicates a situation where no alternative could be made to improve one objective without hindering the others, and is an important concept in the subject of multi-objective optimization. Pareto optimality has been widely applied for alternative selections in the field of engineering and biology. Applications of Pareto optimality include multi-task learning^48^, few-shot learning^49^ and transfer learning^50^. The functions^51^ and evolution^52^ of genes underlying biological system has also been studied using Pareto optimization.

In PMGCN, Pareto optimization was applied to combine the applied layer-specific and multilayer metrics with the aim to improve efficiency of network dismantling. The Pareto front calculation function is developed in Python to compute the Pareto front in multi-metrics optimization. The core idea of the function is to iteratively remove dominated nodes (specifically, if a given node is not worse than any other node across all layers and is strictly better in at least one layer, then the node is considered non-dominated). Ultimately, the function identifies the Pareto-optimal solutions, i.e., those solutions not dominated by any other. The return of the function is a list of indices specifying which nodes are on the Pareto front. This method has a time complexity of $O({n}^{2}d)$, where $n$ is the number of nodes and $d$ is the dimensionality of the metric function.

#### Failure Cascading approach for the dismantling of Multi-Disease Network

Greedy approaches preferentially removing the node on the Pareto front or of the top ranking, are iteratively applied during key node removal procedures until the multiplex network was fragmented into non-extensive disconnected clusters. It features that the size of the Largest Mutually Connected Component (LMCC) of the network will gradually decrease and suddenly reduced to $O({n}^{1/2})$. The gene sequence $S$ was subsequently obtained.

In the case of one key node removed from one layer of the multiplex network, a failure cascading approach for the key node is then adopted for the multilayer structure (Supplementary Fig. 2b). In brief, once one node is removed, the connected components of all the layers in the multiplex network are recomputed; and only links between nodes that belong to the same connected component in all layers were reserved. Via this approach, we iteratively identify a parsimonious set of genes that are important for both of the two disease layers.

### Metrics on chronic inflammatory disease datasets

In order to evaluate the performance of the PMGCN algorithm in predicting chronic inflammatory diseases, we adopted three evaluation metrics, namely AUC (Area Under the Curve), F1-score, and ACC (Accuracy). The formula of the metrics is listed below:

F1 score,

9 $${{{\rm{F}}}}1\,{{{\rm{Score}}}}=2\times \frac{{{{\rm{precision}}}}\times {{{\rm{recall}}}}}{{{{\rm{precision}}}}+{{{\rm{recall}}}}}=\frac{{TP}}{{TP}+\frac{1}{2}({FP}+{FN})}$$

Accuracy,

10 $${{{\rm{Accuracy}}}}=\frac{{TP}+{TN}}{{TP}+{TN}+{FP}+{FN}}$$

where *TP* is the number of true-positive calls, *FP* is the number of false-positive calls, and *FN* is the number of false-negative calls,

#### Marker combinations

Contour plots were generated using *Seaborn (v0.12.2)* to visualize the complementarity of biomarkers, and based on the observations we created five biomarker panels as follows:

1. ComKey: this set contains the gene sequence for the optimal percolation algorithm which fragments the network with the smallest number of nodes $\widetilde{n}$.
2. ComDEG: this set contains the top $\widetilde{n}$ common differentially expressed genes (genes with adj.P < 0.05 for both diseases were ordered by $\left|{\log }_{2}{FC}\right|$ of the chronic inflammatory disease) between periodontitis and one chronic inflammatory disease.
3. DEG: this set contains the top $\widetilde{n}$ differentially expressed genes (genes with adj.P < 0.05 were ordered by $\left|{\log }_{2}{FC}\right|$) for the chronic inflammatory disease.
4. ComKey+ComDEG: A set containing genes of ComKey and ComDEG.
5. ComKey+DEG: A set containing genes of ComKey and DEG.

#### Random Forest Model

The performance of the biomarkers in predicting chronic inflammatory diseases was assessed using Random Forest classifiers. Random Forest (RF) models were created using *scikit-learn (v1.5.2)*. The number of estimators was set to 200, other parameters were kept as default.

#### Bootstrapping with 5-fold stratified cross validation

To evaluate the performance of RF models in predicting chronic inflammatory diseases, we performed a stratified 5-fold cross validation. To assess the stability of the model performance metrics (AUROC, F1-Score, and Accuracy), we combined 5-fold cross-validation with bootstrap resampling. For each of the 5 folds, we created 200 bootstrap replicates by sampling with replacement from the fold’s training set. A Random Forest model was trained on each bootstrap sample and validated on the fixed test fold of the cross-validation. The resulting 1000 performance estimates (5 folds × 200 replicates) for each metric were visualized in boxplots (*seaborn v0.13.2*) to illustrate their distributions and robustness.

#### Label permutations

Empirical null distributions were created through label permutations. For each bootstrap sample, the label permutated counterparts were generated, and the same computational framework was performed. The performance metrics (AUROC, F1-Score and Accuracy) of the RF models were visualized using boxplots (*seaborn v0.13.2*).

### scRNA-Seq data analysis

#### Data preprocessing and quality control

We obtained five single-cell RNA-seq datasets (Supplementary Table 2) and preprocessed the expression data using the *Seurat* package (*v5.0.3*). For all the UC datasets, we applied consistent quality control criteria: keeping cells with detected gene numbers between 200 and 4000, total UMI counts below 50,000, mitochondrial gene percentage below 30%, hemoglobin gene percentage below 5% and ribosomal gene percentage below 60%. 177,284 cells were retained after quality control.

For the periodontitis dataset, similar preprocessing was applied with dataset adjusted thresholds for quality control: detected gene numbers between 200 and 5000, total UMI counts below 50,000, mitochondrial gene percentage below 15%, hemoglobin gene percentage below 5% and ribosomal gene percentage below 40%. 11,038 cells were retained consequently. The dataset-specific thresholds were tuned to account for variations between the independently generated datasets, ensuring that quality control was appropriate for each context to achieve biologically meaningful clusters.

#### Cell clustering

Unsupervised cell clustering was performed on the processed cell expression data for UC datasets using Seurat pipeline, as follows: (1) normalization of raw UMI counts matrix was performed using *SCTransform* (*v0.4.1*) with default parameters, (2) principal component analysis (PCA) performed by using RunPCA functions with default parameters, (3) batch effect correction was performed by the *Harmony* (*v1.2.3*), (4) unsupervised clustering was performed by using the FindNeighbors (reduction = “harmony”, dims = 1:20) and FindClusters (resolution = 0.5 and other default parameters) functions, (5) data visualization of cell clusters was performed using UMAP with the top 20 Harmony-corrected principal components (PCs).

For the periodontitis dataset, the procedures are as follows: (1) normalization of raw UMI counts matrix for periodontitis dataset was performed using NormalizeData function with default parameters, (2) finding the top 2000 highly variable genes with default parameters, (3) principal component analysis (PCA) performed by using the ScaleData and RunPCA functions with default parameters. Steps 4 and 5 were the same for processing the UC datasets.

#### Annotation of cell clusters

Cell types were manually annotated using canonical marker genes^53,54^. Key markers are listed below:

T/NK cells: CD3D, CD3G, NKG7

B cells: CD79A, CD19

Plasma cells: IGHA1, IGHA2

Endothelial cells: VWF, PECAM1

Epithelial cells: EPCAM (UC) or HOPX, IGFBP5, LAMB3 (periodontitis)

Myeloid cells: LYZ

Fibroblasts: COL1A1, COL1A2, LUM, CXCL14, ADAMDEC1

Myofibroblasts: RGS5, ACTA2

Glial: S100B.

#### Gene expression visualization and functional analysis

Expression of PMGCN genes (e.g., FOSB) and marker genes in cell clusters were visualized using DotPlot. Positive cells with regard to a key gene were defined as the expressions of the key gene are above 0, proportions of key gene positive cells were calculated against the number of cells for each sample, and *P* values of the cell proportions between disease states were calculated using Wilcoxon rank-sum tests with FDR correction. Violin plots combined with boxplots were applied to visualize expression distributions (*ggplot2 v3.4.2*).

#### Sub-clustering and annotation of epithelial cell and B cell subpopulations

The expression data of epithelial cells and B cells were further obtained, processed, sub-clustered and annotated. The canonical biomarkers for the sub-clusters of epithelial cells and B cells are listed below:

Epithelial cells:

Proliferating epithelial cells (TA): MKI67, TOP2A, PCNA

Early colonocytes: CA2, SLC26A2, FABP1

Intermediate colonocytes: B3GNT7, ABR, ADH1C, STEAP3

Mature colonocytes: AQP8, GUCA2A, CEACAM1

BEST4/OTOP2 cells: BEST4, OTOP2, CA7

LND cells: LCN2, NOS2, DUOX2

Goblet cells: CLCA1, MUC2, TFF3

Enteroendocrine cells: CHGA, NEUROD1

Tuft cells: POU2F3, TRPM5

B cells:

Follicular B: IGHD, TCL1A, FCER2

Memory B: CD27, CD44

Cycling B: MKI67

#### Functional analysis of DEGs in cell subpopulations

Differentially expressed genes (DEGs) between positive groups and the corresponding negative group were identified using FindMarkers ( | log2FC | > 1, FDR < 0.05). GO/KEGG enrichment analysis was performed on DEGs using clusterProfiler (*v4.6.2*).

#### Single-cell Pseudotime

Differentiation trajectory was constructed using *Monocle3* (*v1.3.7*). PCA (dim=50) and UMAP were applied, followed by batch correction via mutual nearest neighbor alignment. Louvain clustering defined cell states, and trajectories were rooted at stem cells (LGR5^+^, OLFM4^+^). Trajectory-dependent genes (Moran’s *I* > 0.25) were filtered to exclude ribosomal (RPL/RPS) and mitochondrial (MT-) genes. Dynamic modules were identified via *k*-means clustering (*k* = 4), visualized in pseudotime heatmaps (*ClusterGVis v0.1.2*), and functionally annotated using GO/KEGG enrichment (*clusterProfiler v4.6.2*).

#### Ethical consideration

No ethical approval was sought/required for this study as the genetic information accessed/used was de-identified and publicly available.

## Results

### A multi-disease network percolation framework to identify omic biomarkers for UC

**Fig. 1:**
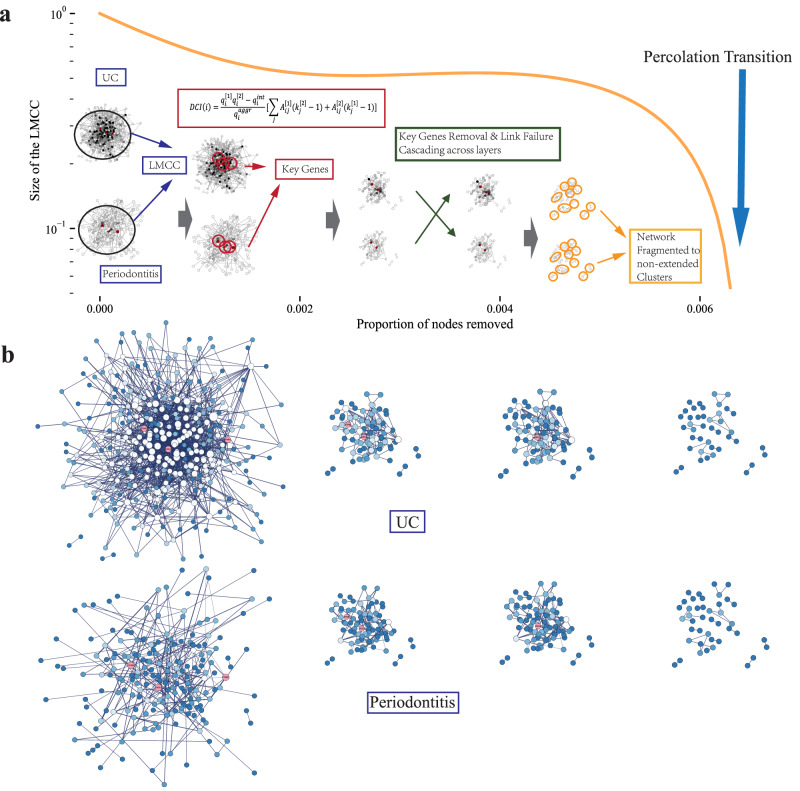
PMGCN identifies omic biomarkers for ulcerative colitis (UC) using optimal percolation on multi-disease networks. **a** Schematic illustration of the optimal percolation dynamics in the periodontitis and UC multiplex network. The X-axis represents the proportion of nodes removed, and the Y-axis which is displayed using a logarithmic scale corresponds to the size of the LMCC. **b** Visualization of multiplex network topological reorganization through iterative depletion of key genes. Corresponding panels demonstrate phase transition dynamics: intact networks (left) maintaining small-world architecture with modular interconnectivity transition to disintegrated states (right) following percolation-based node ablation.

PMGCN is a dedicated computational framework that aims to facilitate omic biomarker discovery regarding chronic inflammatory diseases by harnessing the associations with Periodontitis. PMGCN first constructed a multi-disease network for periodontitis and UC in the form of a multiplex gene co-expression network and then extracted a parsimonious set of key genes as omic biomarkers using optimal percolation approaches. Fig. 1.a and Supplementary Fig. 1 provide an overview of PMGCN’s main components and workflow. In brief, the multi-disease gene co-expression network (Supplementary Table 3), constructed from bulk transcriptomic profiles (Supplementary Fig. 1 and *Methods*) for a Periodontitis cohort and a UC cohort, was initially occupied by the largest mutually connected component (LMCC), and the degree distribution follows power law (Supplementary Fig. 2a). Heuristic optimal percolation algorithms (*Methods*) were applied to rank the nodes, and the top ranked key gene nodes were eliminated sequentially, with each key node deletion causing link failures in one layer to propagate to another layer (Supplementary Fig. 2b and *Methods*). Among the applied optimal percolation method set, Pareto optimality^47^ formed by CoreHD^44^ and DCI^35^ algorithms (Supplementary Data 1 and Methods) achieved a plausible and parsimonious sequence of genes to fragment the LMCC into non-extensive disconnected clusters. During the network attacking, the size of the LMCC decreases gradually initially and plummets to $O({n}^{1/2})$ (here $n$ denotes the number of nodes of the network) when the key nodes have been iteratively attacked, which indicates a percolation transition (Fig. 1a). In the percolation process for the Periodontitis-UC multi-disease network, the gene sequence containing PTGR1, FOSB, and CXCL5 was identified as the key targets and removed one by one. The Periodontitis-UC multi-disease network is dismantled in an efficient way and finally collapses to non-extensive disconnected clusters (Fig. 1b). In addition to the selected hyperparameter configuration (Supplementary Table 3), a range of network parameters were employed to evaluate the parameter sensitivity of PMGCN. Across these varied network configurations, two of the three percolation-derived markers (FOSB and CXCL5) were consistently identified (Supplementary Data 2).

### The PMGCN genes and linking gene modules are associated with inflammatory signaling pathways

**Fig. 2:**
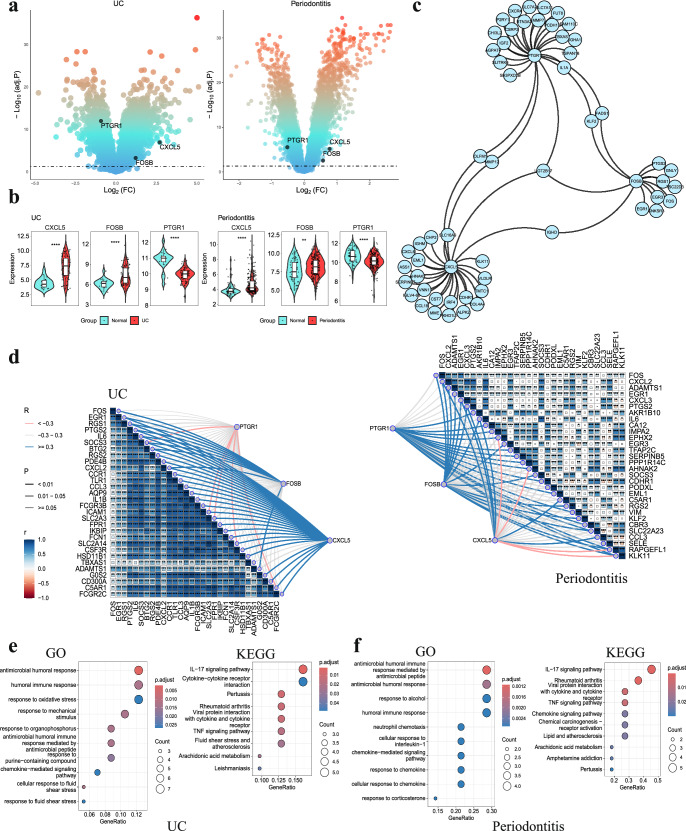
Expression visualization and functional annotations of the key genes and the linking gene set in UC. **a** Comparative transcriptome profiling. Volcano plots show differentially expressed genes in (left) UC and (right) periodontitis. Black points denote the key genes PTGR1 (downregulated), CXCL5 (upregulated) and FOSB (upregulated). Gray dashed lines indicate thresholds (adj.*P* = 0.05). **b** Disease-associated expression visualization of the key genes. Violin plots compare expression levels of the three key genes between healthy controls and disease samples. Center lines show medians, boxes indicate interquartile ranges. Statistical significance was determined by Student’s *t* test, with asterisks indicating significance levels (*P < 0.05, **P < 0.01, ***P < 0.001). **c** UC-specific gene co-expression network linked to the key genes. Network visualization reveals the genes co-expressed with PTGR1, FOSB, and CXCL5. **d** Visualization of Pearson correlations between key genes (PTGR1, FOSB, CXCL5) and their top 30 correlated genes in ulcerative colitis and periodontitis, with a color gradient encoding both correlation magnitude and asterisks indicating significance levels (*P ≤ 0.05, **P ≤ 0.01, ***P ≤ 0.001). Circos-style visualization reveals the correlation patterns between key genes and the top 30 correlated gene set, where cord widths quantifying effect sizes and hues denote directionality (pink: positive *R* > 0.3, blue: negative *R* < −0.3). Functional enrichment of the linking gene set in UC (**e**) and periodontitis (**f**). Dot plot visualizations of GO and KEGG pathways for the linking gene set are presented, displaying the top 10 significant terms (ranked by adjusted *P* values) are shown. Point size reflects gene counts per pathway, while color gradient denotes adjusted *p*-value.

To explore the role of key genes (CXCL5, PTGR1 and FOSB) screened by PMGCN in the two diseases, we observed that CXCL5 and FOSB were significantly upregulated in both periodontitis and UC samples compared to the corresponding control samples, while PTGR1 was downregulated in both periodontitis and UC samples (Fig. 2a, b). CXCL5 (C-X-C Motif Chemokine Ligand 5) is a cytokine belonging to the CXC chemokine family that plays a crucial role in immune response, inflammatory diseases such as ulcerative colitis and periodontitis. Elevated CXCL5 levels contribute to excessive neutrophil infiltration into mucosal tissues, leading to inflammation and tissue destruction^55^. FOSB, a transcription factor in the Activator Protein−1 (AP−1) complex, plays a role in inflammation and immune regulation, making it relevant to chronic inflammatory diseases^56^. In UC, FOSB is involved in inflammatory responses, immune cell and intestinal epithelial cell proliferation and differentiation^57^, whereas FOSB plays a role in inflammation, bone resorption and osteoclast activation during periodontitis^58^. PTGR1 (Prostaglandin Reductase 1) a rate-limiting enzyme involved in the arachidonic acid metabolism pathway, which is involved in immune signaling by inactivation of proinflammatory eicosanoids such as prostaglandins and leukotriene B4. PTGR1 expression is associated with psoriasis, a chronic inflammatory disease^59^. Moreover, the dysregulated prostaglandin signaling can lead to excessive inflammation and mucosal damage in UC^60^. In line with previous, PMGCN genes may serve a vital role in the inflammation responses.

Next, we analyzed the expression of linking genes correlated with the PMGCN key genes. CXCL5, PTGR1 and FOSB have 25, 24 and 12 linking genes in the UC layer, respectively (Fig.2c). Among them, OLFM1 and MMP12 are shared linking genes between CXCL5 and PTGR1, while IGHD is shared between CXCL5 and FOSB. KLF2 and FADS1 are common to PTGR1 and FOSB, and UGT2B17 is the only gene connecting the three key genes (Fig.2c). In contrast, CXCL5, PTGR1 and FOSB only have 3, 5 and 3 linking genes in the periodontitis layer (Supplementary Fig. 3a). The heatmap demonstrates distinct expression patterns of these linking genes between the UC and the control samples, with the majority of the linking genes being upregulated in UC and a small subset downregulated (Supplementary Fig. 3b). Furthermore, correlation plots overview the top correlated genes for the three key genes in Periodontitis and UC, and demonstrate significant and diverse correlations (Fig. 2d).

GO analysis found UC linking genes are enriched in antimicrobial humoral response, humoral immune response, response to oxidative stress (Fig. 2e), while KEGG analysis indicates significant enrichment in IL17 signaling pathway, cytokine-cytokine receptor interaction, rheumatoid arthritis and TNF signaling pathway (Fig. 2e). Further analysis found that periodontitis linking genes are enriched in a similar GO processes such as antimicrobial humoral immune response mediated by antimicrobial peptide, and KEGG pathway such as IL17 signaling pathway and TNF signaling pathway (Fig. 2f). Together, these results suggest that PMGCN genes and linking genes may mediate the dysregulation of inflammatory signaling pathways in regulating inflammation responses in UC and Periodontitis.

### PMGCN-screened CXCL5 targeting fibroblasts promotes UC and Periodontitis progression

To further investigate the potential role of PMGCN key genes in the pathogenesis of UC and periodontitis, we integrated and harmonized 4 published scRNA-seq data including 14 healthy, 23 inflamed UC and 13 non-inflamed UCs, while we re-analyzed oral mucosa scRNA-seq data^53^. After quality control, 177,284 cells from UC datasets and 11,038 cells from periodontitis were retained. The clustering on single cells revealed major cell types within the epithelial compartment, and the immune compartment (T cells, B cells, plasma cells, myeloid cells, cytotoxic T/natural killer cells), and stromal compartment (endothelial, myofibroblasts, fibroblasts and gial) (Supplementary Fig. 4). Cell types were manually curated by known marker genes with epithelial expressing EPCAM, myeloid cells expressing LYZ, T/NK cells expressing NKG7, CD3D and CD3G, B cells expressing CD79A and CD19, plasma cells expressing IGHA1 and IGHA2, endothelial expressing PECAM1 and VWF, fibroblast expressing LUM, ADAMDEC1, CXCL14, and myofibroblast expressing ACTA2 and RGS5. Cellular composition within the stromal compartments including endothelial and myofibroblasts differed significantly between healthy/non-inflamed UC and inflamed UC (Supplementary Fig. 4d). Then, we found that PMGCN key genes CXCL5, FOSB and PTGR1 are expressed in various cell clusters (Supplementary Fig. 5).

**Fig. 3:**
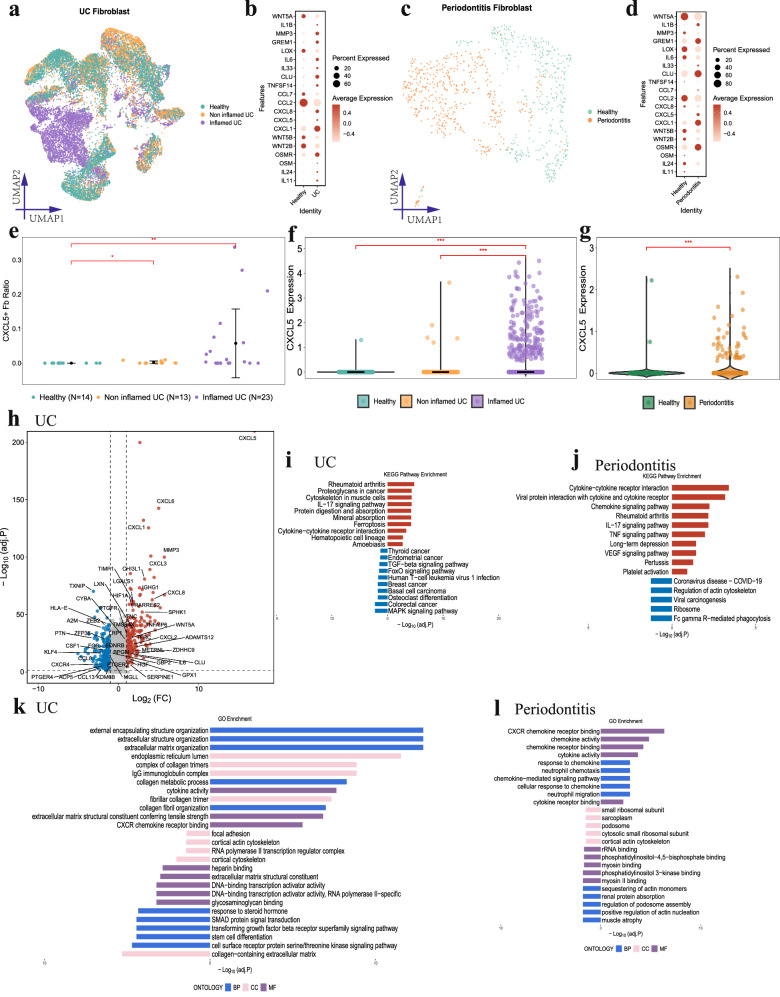
CXCL5 expression and the affected pathways in fibroblast cells of UC and Periodontitis. **a,c**, UMAP visualization of fibroblast cells from the UC (**a**, *N*_fibroblast_ = 16,737 cells) and periodontitis (**c**, *N*_fibroblast_ = 867 cells) single-cell dataset, with color-coding indicating disease states of cells. **b**, **d** The comparative dotplot visualization displays expression of markers of inflammatory and activated fibroblasts in the UC (**b**) and periodontitis (**d**) single-cell datasets. **e** Ratio of CXCL5^+^ fibroblasts in healthy, non-inflamed, and inflamed UC samples. The distributions of ratio indicate a notable increase in the proportion of CXCL5-expressing fibroblasts in UC. *P* values were determined by two-sided Wilcoxon rank-sum test, with asterisks indicating significance levels (*P < 0.05, **P < 0.01). **f**, **g** CXCL5 expression levels in fibroblast in UC (healthy vs. non-inflamed vs. inflamed UC) (**f**) and periodontitis (healthy vs. periodontitis) (**g**). *P* values were determined by Wilcoxon rank-sum test, with asterisks indicating significance levels (***p < 0.001). **h** Volcano plots display differentially expressed genes (adj.*P* < 0.05 & |log_2_FC | > 1) of fibroblast cells (CXCL5^+^ vs. CXCL5^-^) of the UC single-cell datasets, with inflammation-associated genes (Gene Ontology Term No.0006954: inflammatory response) highlighted. **i**, **j** KEGG pathway enrichment plots illustrate top 10 significantly up-regulated (red) and downregulated (blue) pathways for the differentially expressed genes of fibroblast cells (CXCL5^+^ vs. CXCL5^-^) of the UC (**i**) and periodontitis (**j**) single-cell datasets. **k**, **l** GO pathway enrichment plots display top significantly up-regulated (the right bars) and downregulated (the left bars) pathways for the differentially expressed genes of fibroblast cells (CXCL5^+^ vs. CXCL5^-^) of the UC (**k**) and periodontitis (**l**) single-cell datasets. For each category of GO term (BP: biological process; CC: cellular components; MF: molecular function), the top 5 significant terms were selected.

We found disease-specific fibroblasts upregulated inflammatory gene signatures^61^ compared with their healthy counterparts (Fig. 3a–d) in the UC and oral cavity. Notably, CXCL5 is mainly enriched in fibroblasts and significantly increased in the fibroblasts of active UC patients and periodontitis (Fig. 3e–g). In periodontitis, gingival mucosa fibroblasts are enriched in the expression of inflammatory genes, particularly those involved in recruiting neutrophils (CXCL1, CXCL2, CXCL6 and CXCL8) to aid in wound healing^62,63^. Functional analysis of genes upregulated CXCL5^+^ fibroblasts compared to CXCL5- fibroblasts found that they were enriched in cytokine activity, CXCR chemokine receptor binding in GO (Fig. 3k, l), and cytokine-cytokine receptor interaction and IL17 signaling pathway in KEGG (Fig. 3i, j). In line with previous study^64^, our PMGCN screened CXCL5 as the core gene linking UC and periodontitis, which exhibited that the inflammatory fibroblast in the intestines arises in inflammatory environments similar to inflamed gingival mucosa.

### Downregulation of PTGR1 in epithelial compartments in UC

**Fig. 4:**
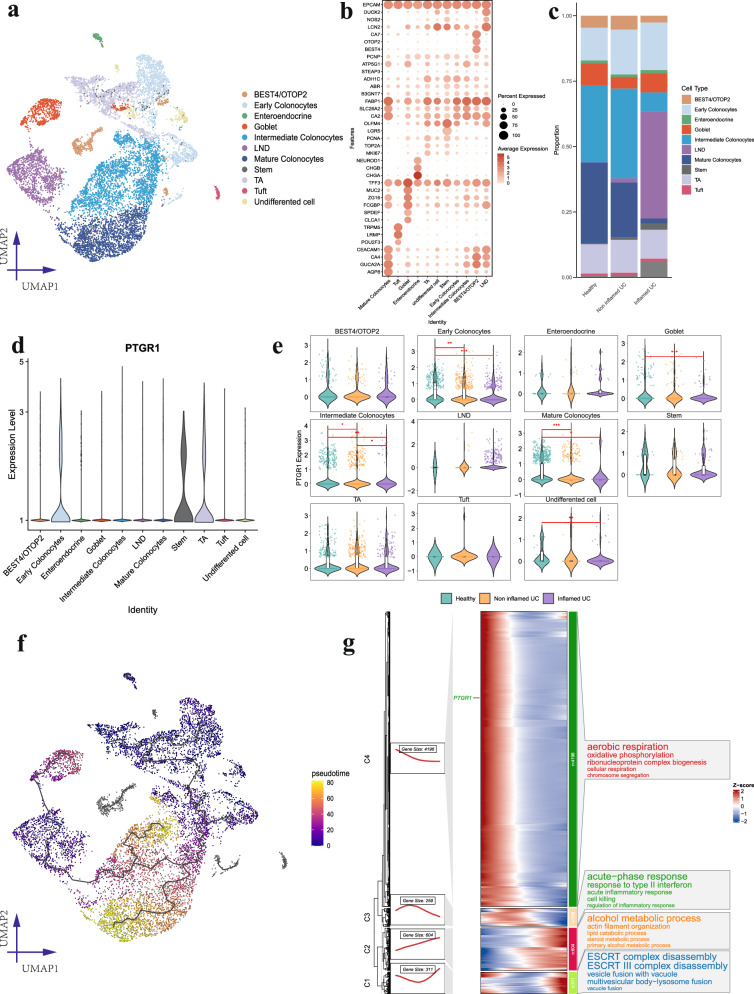
Single-Cell transcriptomic profiling of UC epithelial cell reveals subpopulation heterogeneity and PTGR1-associated dynamics. **a** UMAP visualization of epithelial cell heterogeneity (*N*_epithelial_ = 20,864 cells) from UC single-cell RNA-seq datasets. Distinct cell clusters were annotated into 11 epithelial subtypes based on canonical marker genes. **b** Dot plot illustrates canonical marker genes expression patterns for the 11 epithelial subpopulations, demonstrating unique transcriptional signatures across epithelial compartments. **c** Compositional shifts of cell clusters revealed by stacked bar plot quantification, showing significant expansion of LND cells (purple) in inflamed UC samples compared to healthy controls and non-inflamed UC samples. **d** Violin plots displaying PTGR1 expression across epithelial subtypes, exhibiting elevated levels in stem cells, transit-amplifying (TA) cells, and early colonocytes. **e** Disease-associated PTGR1 dysregulation across epithelial compartments. Violin plots compare PTGR1 expression between healthy, non-inflamed UC, and inflamed UC samples. Asterisks denote significance levels determined using pairwise Wilcoxon rank-sum tests (*P < 0.05, **P < 0.01, ***P < 0.001). **f** UMAP of epithelial cells with colors indicating pseudotime (trajectory computed by Monocle3), and stem cells were treated as the trajectory root. **g** Pseudotime-dependent gene dynamics. k-means clustering (*k* = 4) of trajectory-associated genes (Moran’s *I* > 0.05) identified four expression modules (left panel). Heatmap of Z-score-normalized expressions (rows = genes, columns = cells) displays PTGR1 co-clustered with genes in C4 (middle panel). GO enrichment revealed C4’s association with “aerobic respiration” (adj.*P* < 0.001) and “oxidative phosphorylation” (adj.*P* < 0.001) (right panel).

Next, we examined PTGR1 expression in the epithelial compartments. Deep annotation of epithelial cells revealed cells consist of stem, TA, early, intermediate, and mature colonocytes, BEST4/OTOP2, Goblet cells, Tuft and LND (Fig. 4a,b). Similar to Crohn’s disease^65^, we also observed a dramatic increase in LND cells in inflamed UC samples compared to healthy and non-inflamed UC samples (Fig. 4c). Beyond cellular composition alterations, cell-specific transcriptional changes with disease activity were also observed (Fig. 4e). Notably, we found PTGR1 is mainly expressed in early colonocytes, stem cells and TA cells, while a significant decrease in PTGR1 expression was found in early colonocytes, intermediate colonocytes and mature colonocytes of inflamed UC samples (Fig. 4d, e). A similar trend of PTGR1 expression was observed in non-inflamed UC compared to healthy samples (Fig. 4e). Further analysis found that PTGR1^+^ early colonocytes are positively correlated with oxidative phosphorylation and reactive oxygen species production, but negatively correlated with tight junction, notch signaling pathway and FoxO signaling pathway (Supplementary Fig. 6). Notch and FoxO signaling are known to regulate the proliferation and differentiation of intestinal stem and regulate cell fate choice to control epithelial cell homeostasis^66^. To further investigate whether PTGR1 impacts the development of epithelial cells, we performed trajectory analysis (*Methods*) on epithelial cells. Similar to previous study, stem cells and TA cells had the highest inferred stemness score (Fig. 4f). Heatmap of pseudotime correlated genes found that these genes were distributed to four gene clusters, PTGR1 belonging to C4 cluster enriched in aerobic respiration is correlated with stemness (Fig. 4g). Previous study mentioned that the origins of early LND were strongly associated with early enterocytes^65^. We speculate that PTGR1 may act as an important modulator to suppress early colonocytes differentiation to early LND cells, however, this hypothesis needs further experiments to verify. However, we found that the expression of PTGR1 in oral epithelial of periodontitis is not changed (Supplementary Fig. 4i). These results suggested that PTGR1 may play a role in maintaining the intestine stemness.

### Transcription factor FOSB regulates B cell

In comparison with control subjects, the abundance of mucosal CD19^+^ B cells was significantly increased in inflamed UC and periodontitis (Supplementary Fig. 4c, h). FOSB has several important roles in the immune response, particularly in regulating B cell activation. We found that FOSB^+^ B cells are significantly upregulated in transcription factors such as FOS, NR4A1, NFKBID (Supplementary Fig. 7a, b), due to their importance in cell differentiation and signaling activation^67^. KEGG pathway analysis reveals that the pathways including IL−17 signaling pathway, MAPK signaling pathway, TNF signaling pathway were significantly upregulated in FOSB^+^ B cells for both UC and periodontitis (Supplementary Fig. 7h). We then hypothesized that FOSB + B cells contribute to the pathogenesis of UC and periodontitis.

**Fig. 5:**
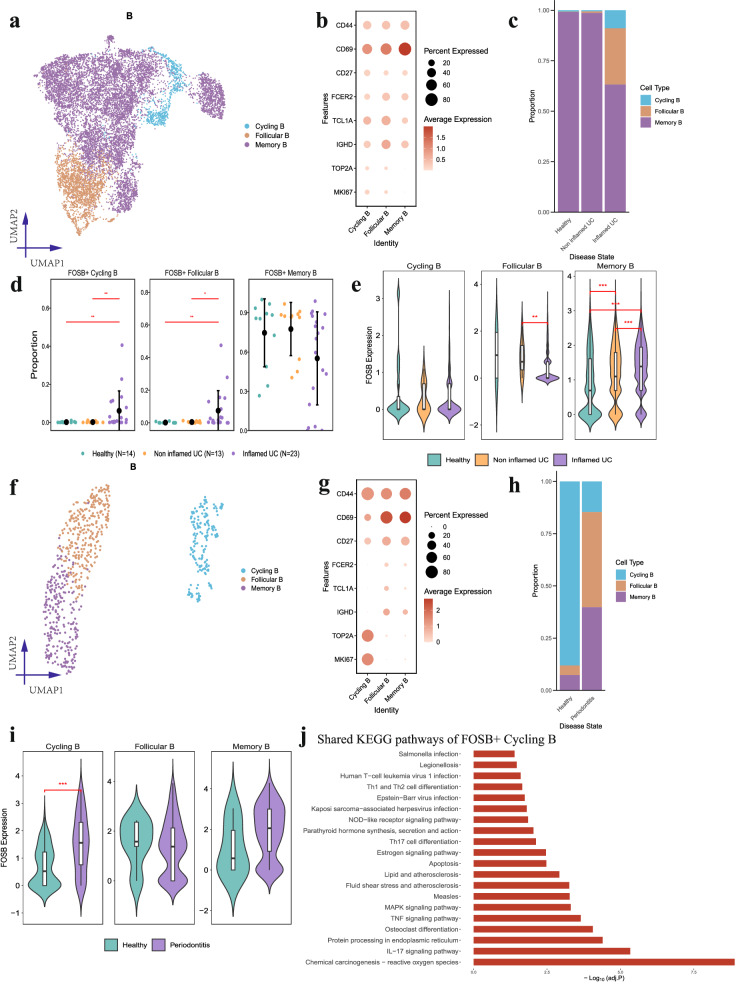
FOSB Expression in B Cell Subpopulations in UC and Periodontitis. **a**, **f** UMAP visualization of B cell subpopulations (i.e. Cycling B, Follicular B, Memory B) in the UC (**a**, *N*_B_ = 12,742 cells) and Periodontitis (**f**, *N*_B_ = 717 cells) single-cell transcriptomic data. **b**, **g**, Dot plot illustrates canonical marker genes expression patterns for the three B cell subpopulations in UC (**b**) and periodontitis (**g**). **c**, **h**, Compositional shifts of B cell subclusters in UC (**c**) and Periodontitis (**h**) datasets revealed by stacked bar plot quantification. **d** Proportions of FOSB^+^ cells in each of the B cell subpopulations in healthy, non-inflamed, and inflamed UC samples. Asterisks denote significance levels determined using two-sided Wilcoxon rank-sum tests (*P < 0.05, **P < 0.01). **e**, **i** Violin plots demonstrating FOSB expression levels across B cell subclusters in UC (**e**) samples and periodontitis samples (**i**), with statistical comparisons between disease states (two-sided Wilcoxon rank-sum test; **P < 0.01, ***P < 0.001). **j** The shared significantly upregulated KEGG pathways enriched in the differentially expressed genes of Cycling B cells (FOSB^+^ vs. FOSB^-^) across UC and periodontitis single-cell datasets. The length of bars denotes the adjusted *P* values for UC.

To test this hypothesis, we identified three subpopulations of CD19^+^ B including follicular B, cycling B and memory B in UC (Fig. 5a, b). Further analysis found that follicular B and cycling B were enriched in inflamed UC (Fig. 5c, d). Moreover, the expression of FOSB was significantly increased in memory B cells in UC, while FOSB expression of cycling B cells are increased in periodontitis (Fig. 5e, i). Further analysis found that FOSB^+^ cycling B cells and FOSB^+^ memory B cells are enriched in the similar KEGG pathways with FOSB + B cells in two diseases (Fig. 5j and Supplementary Fig. 8g). Taken together, these results suggest that transcription factor FOSB displays a pathogenic role in two inflammatory diseases by promoting inflammatory pathways.

### PMGCN identifies biomarkers complementary to CommonDEG/DEG in UC prediction

**Fig. 6:**
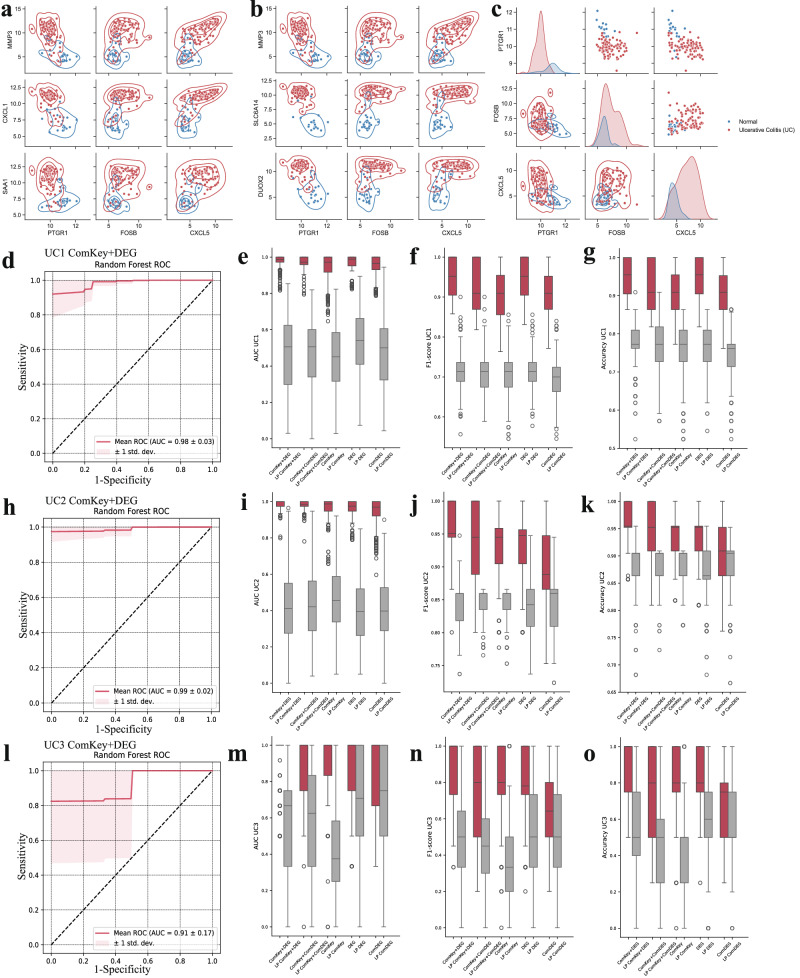
Performance overview of PMGCN identified biomarkers for UC datasets. **a** Pairwise scatter plots integrating ComKey with ComDEG (top 3 commonly differentially expressed genes shared between UC and periodontitis). The multidimensional distribution shows improved separation between disease (red) and control (blue) groups, potentially enhancing model prediction accuracy. **b** Pairwise scatter plots combining ComKey with DEG (top 3 differentially expressed genes in UC). This integration improves group separation in coordinate space, suggesting enhanced discriminatory capability. **c** Pairwise scatter plots and contour plots of ComKey biomarkers (PTGR1, FOSB, CXCL5). Differential PTGR1 expression effectively distinguishes disease status, demonstrating the complementary discriminatory power of this gene combination. **d**–**o** Random Forest classification performance for five biomarker combinations (ComKey + DEG, ComKey+ComDEG, ComKey, DEG, ComDEG) evaluated in three independent ulcerative colitis datasets: UC1 (**d-g**), UC2 (**h**–**k**), and UC3 (**l**–**o**). Left panels show ROC curves with area under the curve (AUC) values for the ComKey + DEG combination. Right panels display boxplots comparing model performance metrics (AUROC, F1-Score, Accuracy) between actual data (red) and permuted labels (gray) across bootstrap iterations with 5-fold cross-validation.

Common differentially expressed genes (ComDEGs) of the interacting diseases were typically referred to as important biomarkers for diseases with associations^13–17^. Therefore, we calculated the ComDEGs (Supplementary Data 3 and Methods) from the Periodontitis cohort and UC cohort and obtained the top 3 ComDEGs for comparable analysis in disease prediction. In addition, differentially expressed genes (DEGs) for the same UC cohort were calculated, and the top 3 DEGs (Supplementary Data 4) were obtained as benchmarks for comparisons. To investigate the strengths and complementarity of ComDEGs, DEGs and PMGCN genes (ComKey) in disease predication, the distribution density of these biomarkers presented by tabulated pair-wise scatter plots offers the visualization of potential pair-wise complementary relations, indicating the potential of forming biomarker panels to improve disease prediction (Fig. 6a–c). Notably, we observed that disease samples and controls with similar FOSB and CXCL5 expressions could be distinguished by PTGR1 expressions (Fig. 6c), which indicates the complementarity within ComKey gene set in disease predictions. Furthermore, PTGR1 expression provides critical discriminative power specifically in samples where ComDEGs and DEGs are expressed at low levels. This confirms that PTGR1 serves as a complementary signal to these gene sets for distinguishing UC from healthy samples (Fig. 6a, b). Thus, we hypothesize that the combined panels of conventional ComDEGs and DEGs with ComKey genes could improve disease predictability.

To further evaluate the predictability of combined panels for UC cohorts, we constructed 5 biomarker panels including ComKey, ComDEGs, DEG, ComKey+CommonDEG, ComKey+DEG and implemented a comprehensive evaluation using three performance metrics, including ROC, F1-score and Accuracy (*Methods*). We then systematically compared the performance of ComKey biomarkers and their combination with differential expression genes (DEGs) against conventional DEG-based approaches (ComDEGs and DEGs) across three independent validation datasets UC1, UC2, and UC3 (*Methods*). Notably, while the UC1 dataset served purposes for network construction (Periodontitis-UC multi-disease network) and initial biomarker panel development, the UC2 and UC3 datasets were specifically reserved for assessing the generalizability of the biomarker panels. To ensure the validity of performance estimation, we employed a nested bootstrapping approach (200 iterations) with stratified 5-fold cross-validation to generate resampled datasets and calculated confidence intervals for each metric. Random Forest models with the same parameter setting were trained on each resampled set to minimize algorithmic bias and evaluated on the corresponding test set.

In the UC1 cohort, the DEG and ComKey+DEG composite panels demonstrated comparable peak performance in median of AUC (0.988 vs 0.986), the median of F1-score (0.951 vs 0.951) and the median of Accuracy (0.954 vs 0.954) (Fig. 6d–g) (*Methods*). The standalone ComKey panel showed marginally lower metrics (AUC: 0.972, F1-score:0.909, Accuracy: 0.909). In the external validation cohorts (UC2 and UC3), the ComKey panel exhibited superior generalizability, achieving improvements over DEG-based approaches in all metrics of AUC (0.986 vs 0.975 in UC2; 1.000 vs 1.000 in UC3), F1-score (0.945 vs 0.948 in UC2; 0.800 vs 0.781 in UC3), and accuracy (0.952 vs 0.952 in UC2; 0.800 vs 0.800 in UC3) (Fig. 6h–o). Remarkably, the ComKey+DEG hybrid panel consistently achieved the highest predictive performance across all external independent tests (median of AUC: 1.000 in UC2, 1.000 in UC3; median of F1-score: 0.951 in UC2, 1.000 in UC3; median of accuracy: 0.955 in UC2, 1.000 in UC3). These results suggest that the synergistic effects for prediction improvement between PMGCN prioritized genes and differentially expressed genes.

In addition, the ComKey genes were also evaluated alongside WGCNA identified genes from UC1 (*Supplementary Methods*), and the results demonstrated that ComKey genes exhibited performance comparable to WGCNA genes across all UC cohorts (Supplementary Fig. 9).

### PMGCN improves predictability for other chronic inflammatory diseases associated with Periodontitis

Expanding beyond ulcerative colitis (UC), we conducted a systematic evaluation of PMGCN’s generalizability across three additional chronic inflammatory diseases with documented periodontitis associations - Alzheimer’s disease (AD), Crohn’s disease (CD) and Parkinson’s disease (PD). PMGCN first constructed multi-disease networks for AD, CD and PD respectively, namely Periodontitis-AD network, Periodontitis-CD network and Periodontitis-PD network:

Periodontitis-AD Network: 440 nodes, 4176 edges.

Periodontitis-CD Network: 446 nodes, 1938 edges.

Periodontitis-PD Network: 401 nodes, 1734 edges.

**Fig. 7:**
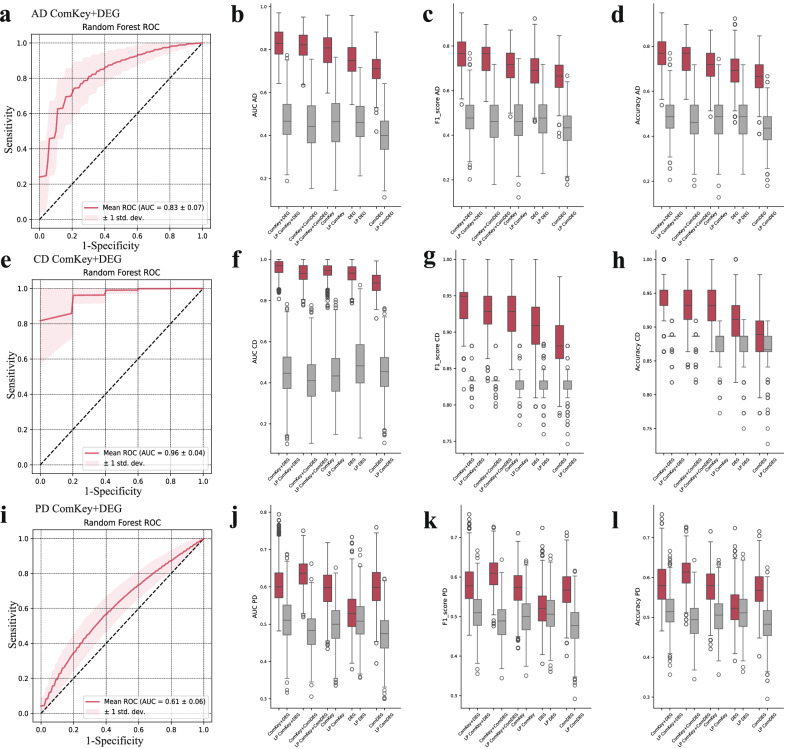
Performance overview of PMGCN for other chronic inflammatory diseases. **a**–**d**, Random Forest classification performance for Alzheimer’s disease (AD). Left panel shows the ROC curve (ComKey+DEG) with AUC value of 0.83 ± 0.07. Right panels display boxplots comparing model performance metrics (AUROC, F1-Score, Accuracy) for all five biomarker combinations (ComKey + DEG, ComKey + ComDEG, ComKey, DEG, ComDEG) between actual data (red) and permuted labels (gray) across bootstrap iterations. **e**–**h**, Classification performance for Crohn’s disease (CD). Left panel shows the ROC curve (ComKey+DEG) with AUC value of 0.96 ± 0.04. Right panels display performance metrics for all biomarker combinations, demonstrating that these biomarker combinations can be successfully generalized to predict Crohn’s disease with high accuracy across multiple performance metrics. **i**–**l**, Classification performance for Parkinson’s disease (PD). Left panel shows the ROC curve (ComKey+DEG) with AUC value of 0.61 ± 0.06. Right panels display performance metrics for all biomarker combinations, showing suboptimal predictive performance for this disease.

PMGCN-derived ComKey biomarkers were identified separately for each individual network topology, with the corresponding biomarkers detailed in Supplementary Data 1, and the optimal algorithm for each disease pair: pareto optimality of CoreHD and EMD for Periodontitis-AD, CI2-DCIz for Periodontitis-CD, and CoreHD-CoreHD for Periodontitis-PD. The ComKey biomarkers were rigorously validated using the same nested bootstrapping approach (200 iterations) with stratified 5-fold cross-validation. Notably, the ComKey sets demonstrated superior predictive performance compared to conventional DEG-based approaches for all disease models:

AD Prediction: AUC improved from 0.749 (DEGs) to 0.808 (ComKey), F1-score increased by 0.026 (0.691 → 0.717), Accuracy enhanced by 0.026 (0.692 → 0.718). (Fig. 7b–d).

CD Prediction: AUC improved from 0.933 (DEGs) to 0.946 (ComKey), F1-score increased by 0.019 (0.909 → 0.928), Accuracy enhanced by 0.021 (0.911 → 0.932). (Fig. 7f–h).

PD Prediction: AUC improved from 0.528 (DEGs) to 0.598 (ComKey), F1-score increased by 0.053 (0.521 → 0.574), Accuracy enhanced by 0.057 (0.523 → 0.580). (Fig. 7j–l).

Moreover, for AD, the combinatorial ComKey+DEG panels achieved peak performance metrics (AUC: 0.828 ± 0.066; F1-score: 0.766 ± 0.072; Accuracy: 0.769 ± 0.070), significantly outperforming the DEG set (paired t-test for AUC, *p* < 0.001, *t* = 37.98, Cohen’s *d* = 1.20) (Fig. 7a–d). And for CD, the combinatorial ComKey+DEG panels achieved peak performance metrics (AUC: 0.969 ± 0.040; F1-score: 0.949 ± 0.028; Accuracy: 0.955 ± 0.025) as well, outperforming the DEG set (paired t-test for AUC, *p* < 0.001, *t* = 20.84, Cohen’s *d* = 0.66) (Fig. 7e–h). For PD, the combinatorial ComKey+ComDEG panel achieved the peak performance metrics (AUC: 0.600 ± 0.057; F1-score: 0.578 ± 0.005; Accuracy: 0.580 ± 0.005), significantly outperforming the DEG set (paired t-test for AUC, *p* < 0.001, *t* = 50.63, Cohen’s *d* = 1.60) (Fig. 7i–l).

To further assess the generalizability of PMGCN-derived biomarkers for CD, an independent external validation cohort (CD2) were obtained. For the CD2 cohort, AUC improved from 0.748 (DEGs) to 0.821 (ComKey), F1-score increased by 0.004 (0.899 → 0.903), Accuracy slightly decreased by 0.001 (0.926 → 0.925). Moreover, the combinatorial ComKey+DEG panel achieved peak performance metrics (AUC: 0.843 ± 0.049; F1-score: 0.903 ± 0.012; Accuracy: 0.928 ± 0.011), outperforming the DEG set (paired t-test for AUC, *p* < 0.001, *t* = 46.93, Cohen’s *d* = 1.48) (Supplementary Fig. 10). Notably, ComKey derived biomarker panels demonstrated significant advantages in all the investigated external validation cohorts.

In addition, ComKey genes were also evaluated alongside WGCNA identified gene sets (*Supplementary Methods*) for the AD, CD, and PD cohorts, and the results demonstrated that ComKey exhibited equivalent or superior performance across all metrics compared to WGCNA gene panels (Supplementary Fig. 11).

## Discussion

In this study, we presented PMGCN, a computational framework for discovering biomarkers for chronic inflammatory diseases, by leveraging the prior knowledge of multi-disease correlations. Traditionally, the exploration of omic biomarker relies on some form of global univariate significance test based on differential expression analysis for single disease^68^. PMGCN complements and supplements the traditional strategy of evaluation omic biomarkers based on single disease cohort and highlights the importance of considering the multi-disease associations for biomarker discovery.

A key innovative feature of PMGCN is the integration of applying multiplex networks to represent multi-disease correlation and optimal percolation algorithms on multiplex networks to prioritize gene expression signals for biomarker identification which leverages the multi-disease associations. PMGCN quantifies the node importance via optimal percolation algorithms for multiplex networks, therefore, the biomarker selection for the four chronic inflammatory diseases was shifted by the corresponding Periodontitis layer, which resulted in gene node sequences that are vital for both two associated diseases. Unlike conventional node removal approaches that assess individual node importance^69–71^, our method collectively applied several heuristic optimal percolation algorithms incorporating Pareto optimality to identify the minimal set of genes required to fragment the multiplex network into disconnected components. For each disease pair, the specific algorithm which outputs the minimum number of vital coding genes was selected. Considering the selected gene sets based on PMGCN are vital for maintaining the global connectivity of the constructed multiplex GCNs, these genes collectively exert maximal influence on the network^46^ and may improve the power for disease prediction. This was partially supported by disease prediction analysis for several chronic inflammatory diseases. Although the PMGCN approach was initially applied to disease pairs between periodontitis and four related chronic inflammatory diseases, it holds potential for extension to disease pairs within the broader class of chronic inflammatory diseases.

Applying PMGCN to periodontitis and UC, the three vital genes (CXCL5, PTGR1 and FOSB) were associated with inflammatory diseases progression. Recent study found that inflammatory fibroblasts in the intestine state only arises in severe inflammatory environments similar to inflamed gingival mucosa^64^. Notably, our study also implicates CXCL5 expression in fibroblasts as playing a vital role in linking periodontitis and UC pathology. PTGR1 is a rate-limiting enzyme involved in the arachidonic acid metabolism pathway and mainly responsible for the deactivation of some eicosanoids, including prostaglandins and leukotriene B4^59^. Previous study mentioned that the origins of early LND were strongly associated with early enterocytes^65^. We found that PTGR1 may have an impact on the intestinal epithelial differentiations in UC, the role of PTGR1 in LND differentiation needs further experiments to verify. While, transcription factors such as FOSB, FOS, NFKIBA, are highly expressed in IGHA1-expressing plasma cells from colitis and IBD patients^72^. Similarly, we also found that the increasing expression and proportion of FOSB in follicular B and memory B cells in the UC patients. FOSB^+^ memory B cells enriched in IL−17 signaling pathway, TNF signaling pathway and MAPK signaling pathway in both diseases, suggesting that FOSB^+^ memory B cells display a vital role in UC and periodontitis. Notably, when these three genes were reranked using conventional differential expression analysis, none were positioned within the top 100 candidates. Moreover, parameter sensitivity analysis confirmed that PMGCN maintains stable biomarker identification across varying network construction settings. These underscore the specific association of CXCL5, PTGR1, and FOSB with percolation dynamics rather than differential expression, demonstrating the value of PMGCN in uncovering biologically relevant signals that conventional methods may overlook.

However, this study has several limitations. First, due to the availability of data, gene co-expression networks for periodontitis and UC were constructed based on microarray datasets generated by different studies, potential confounding factors might exist. Second, due to the small sample size for the periodontitis and UC datasets, the construction of GCNs was performed using differentially expressed genes with a relaxed thresholding, which might lead to false discoveries in edge inference. Moreover, further validation and studies in large prospective cohorts with UC are warranted to confirm the discovery of our method and to optimize the approach.

Overall, PMGCN proves to be a valuable tool for omic biomarker identification in periodontitis-associated chronic inflammatory diseases via applying multiplex network representation and network percolation approaches. By leveraging multi-disease associations, PMGCN identifies key biomarkers and offers new insights into the complex biological interactions that link these conditions.

## Supplementary information

Transparent Peer Review file

Supplemental Information

Description of Additional Supplementary files

Supplementary Data 1

Supplementary Data 2

Supplementary Data 3

Supplementary Data 4

Supplementary Data 5

## Data Availability

All the data used in the study are publicly available via the original publications and the Gene Expression Omnibus (GEO, https://www.ncbi.nlm.nih.gov/geo/). The specific bulk omics datasets used are: periodontitis microarray dataset (GSE16134), UC microarray datasets (UC1: GSE87466; UC2: GSE59071; UC3: GSE48958), AD microarray dataset (GSE132903), CD microarray dataset (CD1: GSE186582; CD2: GSE112366) and PD microarray dataset (GSE99039). The specific scRNA-seq datasets used are: periodontitis scRNA-seq dataset (GSE152042) and UC scRNA-seq datasets (GSE282112, GSE114374, GSE116222, GSE182270). The data used to create the figures is available in the Supplementary Data 5 file. The processed data matrices have been deposited at Zenodo: 10.5281/zenodo.18953072.

## Code availability

The R and Python code written for the PMGCN workflow are available on Github^73^(https://github.com/felixshiyong/PMGCN).
