# Supplementary material for: Identifying omic biomarkers for chronic inflammatory diseases associated with periodontitis using percolation on multi-disease gene co-expression networks
Source: Commun Med (Lond). 2026 Apr 21;6:351. doi: 10.1038/s43856-026-01591-w (PMC13284180; doi:10.1038/s43856-026-01591-w)
Supplement: Supplementary file 2 — Supplemental Information [file 43856_2026_1591_MOESM2_ESM.pdf]

Supplementary Tables:

Supplementary Table 1 | The bulk microarray datasets

Disease	Abbreviations	Tissue	Dataset ID	Case Samples (n)	Control Samples (n)	Total Samples (n)
Periodontitis		Gingival tissue	GSE16134	241	69	310
Ulcerative Colitis	UC1	Mucosal biopsies	GSE87466	87	21	108
Ulcerative Colitis	UC2	Mucosal biopsies	GSE59071	97	11	108
Ulcerative Colitis	UC3	Mucosal biopsies	GSE48958	13	8	21
Alzheimer's Disease	AD	Middle temporal gyrus	GSE132903	97	98	195
Crohn's Disease	CD1	Ileal mucosal biopsies	GSE186582	196	25	221
Crohn's Disease	CD2	Ileal mucosal biopsies	GSE112366	362	26	388
Parkinson's Disease	PD	Whole blood	GSE99039	205	233	438

Supplementary Table 2 | The single-cell RNA sequencing datasets

Disease	Tissue	Dataset ID	Case Samples (n)	Control Samples (n)	Total Samples (n)
Ulcerative Colitis	Gut biopsies (cell atlas)	GSE282112	22	6	28
Ulcerative Colitis	Colonic mesenchyme cells	GSE114374	2	2	4
Ulcerative Colitis	Intestinal epithelial cells (colon biopsies)	GSE116222	6	3	9
Ulcerative Colitis	Plasma cells from colonic biopsies	GSE182270	6	3	9

Periodontitis	Gingival tissue cell populations	GSE152042	2	2	4

Supplementary Table 3 | Parameters and network properties of the Multi-Disease Gene Co-expression Networks.

Disease Pair	Hyperparameters (ρ, α)	Gene Number G_{shared}	Layer	Network Structure (V, E)
P-UC	(0.09,0.95)	475	P	(204,191)
			UC	(332,1003)
P-AD	(0.15,0.85)	440	P	(215,246)
			AD	(374,1842)
P-CD	(0.1,0.9)	446	P	(262,293)
			CD	(330,676)
P-PD	(0.35,0.8)	401	P	(235,387)
			PD	(264,480)

P: Periodontitis. V: Vertex. E: Edge. UC: ulcerative colitis. AD: Alzheimer's disease. CD: Crohn's disease. PD: Parkinson's disease.

Supplementary Figures:

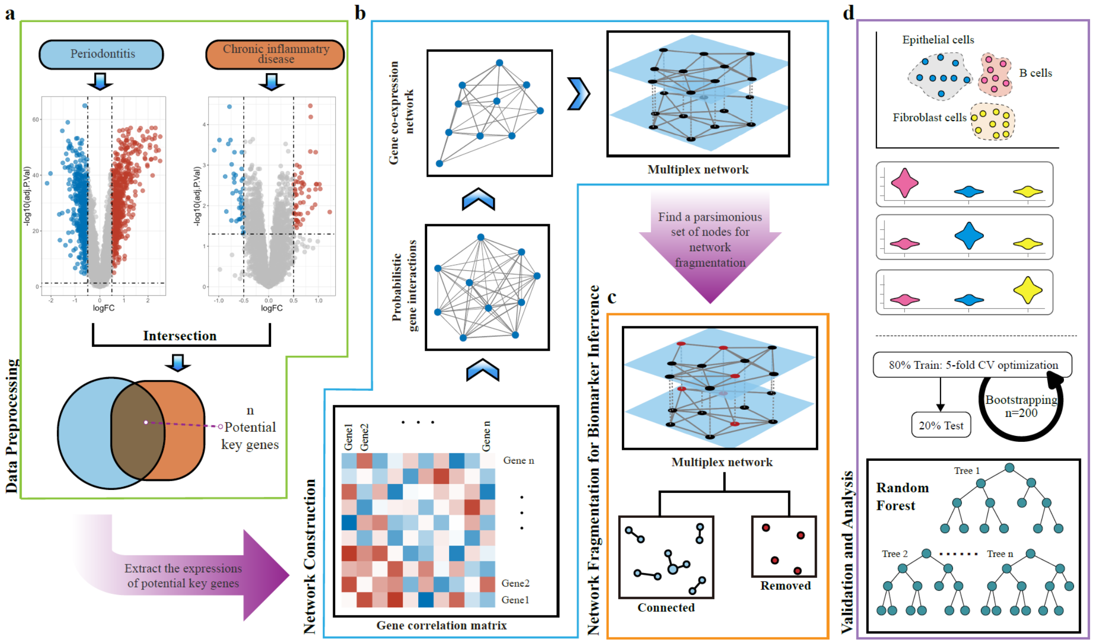

Supplementary Fig. 1. Workflow of PMGCN framework. a, Data preprocessing and candidate gene selection. Differentially expressed genes shared between periodontitis and one chronic inflammatory disease were selected as potential key genes for downstream analysis, based on predefined selection criteria (**Methods**). **b,** Multiplex network construction. Disease-specific gene co-expression networks were computed based on selected potential key gene set and further integrated into a multiplex architecture. **c,** Network based biomarker identification. Minimum biomarker gene sets were determined through optimal percolation algorithms, systematically fragmenting the multiplex network while minimizing node removal. **d,** Biological validations. Marker genes were functionally characterized via single-cell RNA sequencing (scRNA-seq) data analysis, and its performance for disease prediction were evaluated based on a nested bootstrapping approach with the stratified 5-fold cross-validation using random forest classifiers (**Methods**).

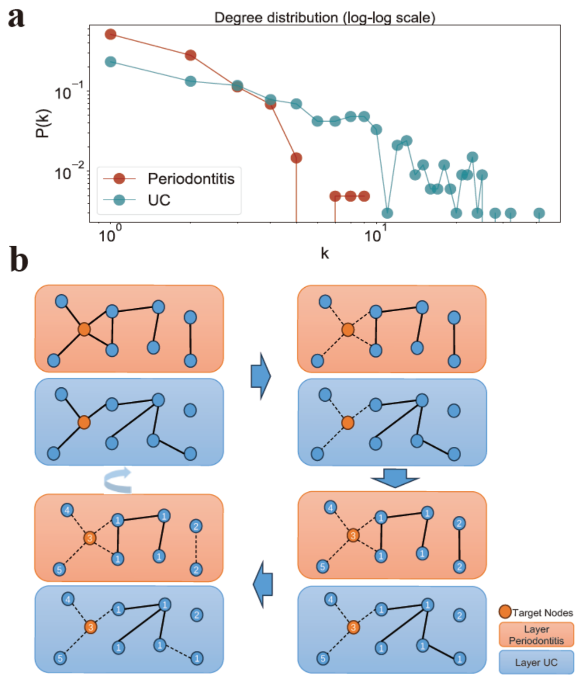

Supplementary Fig. 2. Gene co-expression network degree distributions and network failure propagation. a, Degree distribution of the gene co-expression networks for the periodontitis and UC samples. The x-axis represents the node degree (*k*), and the y-axis shows the proportion of nodes (*P(k)*). Data are displayed on a log-log scale, demonstrating power-law distributions. **b,** Schematic diagram illustrating the node failure cascading procedures. Initial failure nodes are shown in orange, with dashed lines indicating edges to be removed. Numbers denote the index of the connected components.

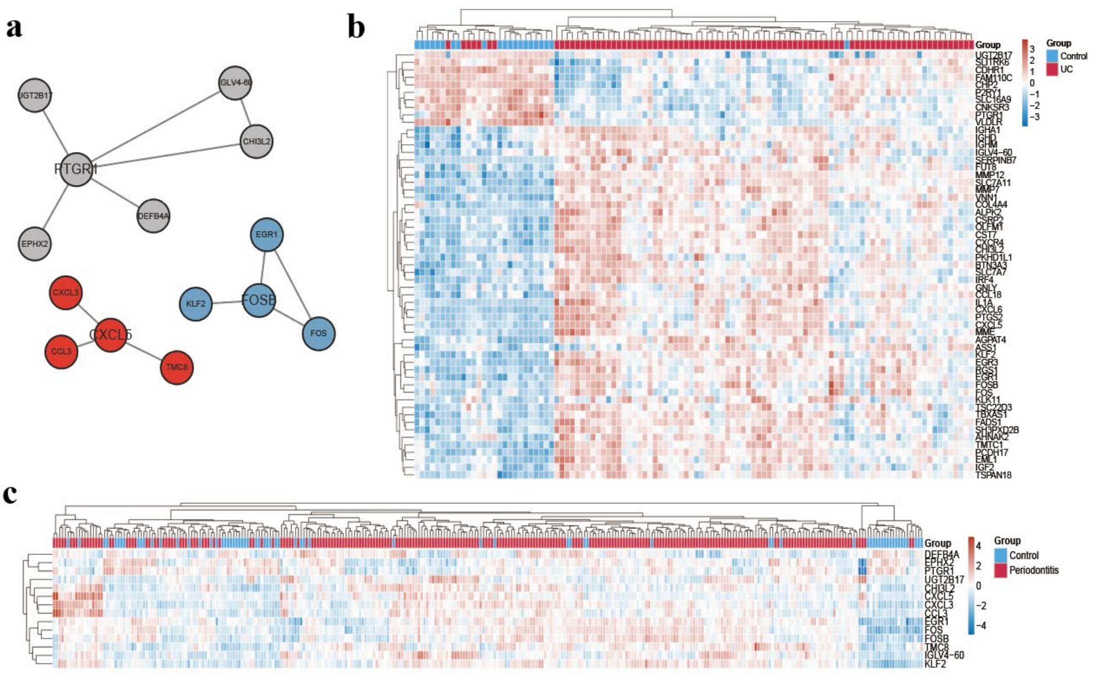

Supplementary Fig. 3. Expression visualization of the key genes and the linking gene set. a, Periodontitis-specific gene co-expression network linked to the key genes. Network visualization reveals the genes co-expressed with PTGR1, FOSB, and CXCL5 in the periodontitis gene co-expression network. **b, c,** Heatmaps of the linking genes for the UC (**b**) and periodontitis (**c**) datasets. Heatmap visualization displays the z-score normalized expression for the linking genes. Columns represent samples grouped by hierarchical clustering (Euclidean distance). Top annotation bars: healthy (blue) vs UC/periodontitis (red).

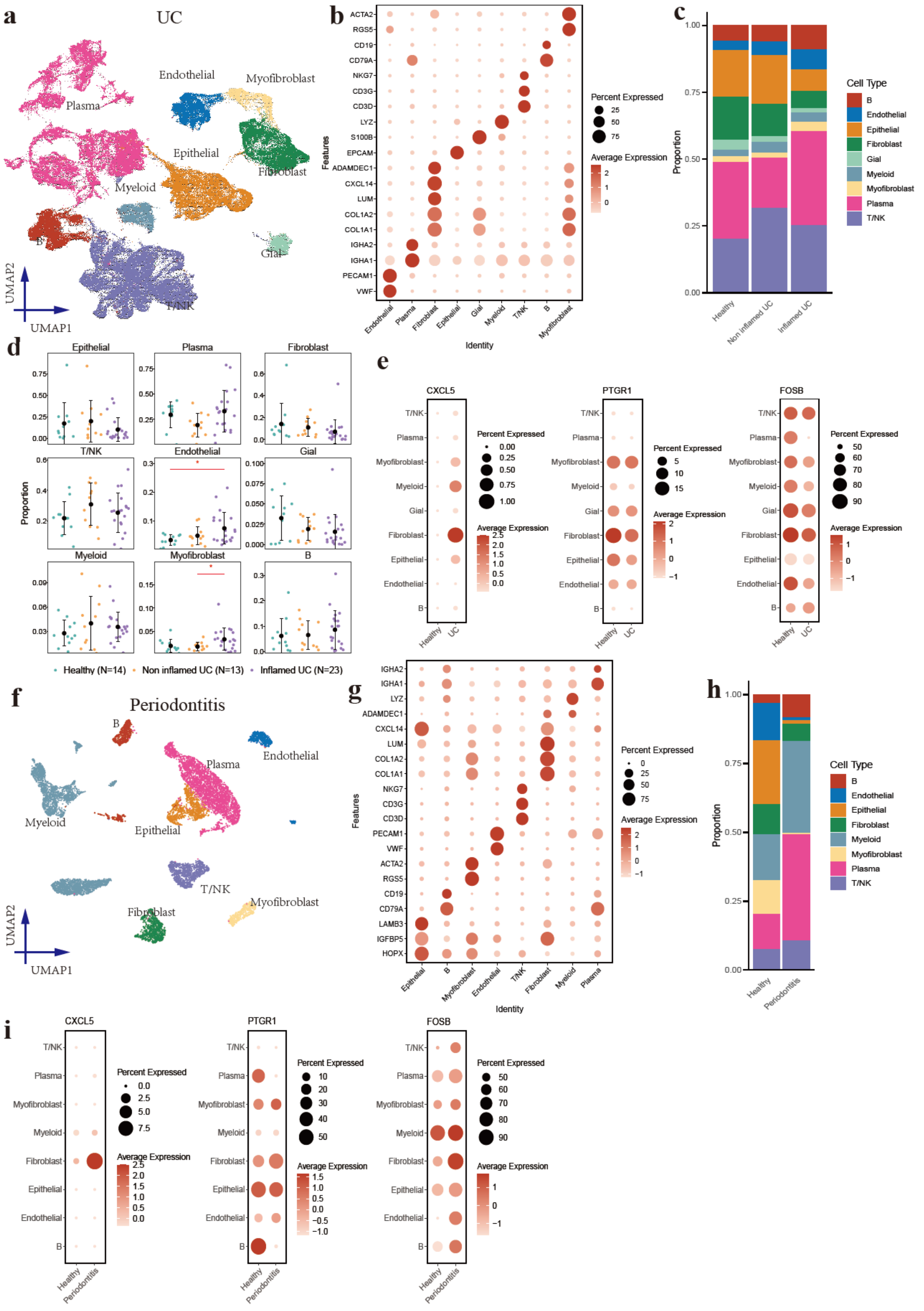

Supplementary Fig. 4. Single-cell transcriptome profiling and comparative expression analysis of the key genes in UC and periodontitis. a, Single-cell transcriptome landscape of ulcerative colitis (UC) displayed through UMAP visualization, with distinct clusters annotated as major cellular populations. **b,** Corresponding dot plot demonstrates cell type-specific expression patterns of canonical

marker genes for the integrated UC dataset, validating cluster identities. **c,** Compositional analysis of cell type proportions across healthy controls, non-inflamed, and inflamed UC groups, visualized as stacked bars. **d,** Scatterplots illustrate cell type proportions in healthy control (N=14), non-inflamed UC (N=13), and inflamed UC (N=23) samples, with statistical significance determined by two-sided Wilcoxon rank-sum test (*P<0.05). **e,** Comparative expression profiles of CXCL5, PTGR1, and FOSB across epithelial and immune cell subsets in healthy versus UC samples, revealing cell population-specific differential expression patterns. Elevated CXCL5 in Fibroblast cells and FOSB in B cells suggest disease-specific activation. **f,** UMAP projection of gingival single-cell profiles in periodontitis, annotated by cell type marker genes. **g,** Dot plot validating cell cluster annotations through established marker genes. **h,** Compositional analysis of cell type proportions across periodontal healthy control and periodontitis groups, visualized as stacked bars. **i,** Dot plots displaying expressions of CXCL5, PTGR1, and FOSB across cell types in healthy versus periodontitis conditions. Consistent with UC, elevated CXCL5 in Fibroblast cells and FOSB in B cells were observed.

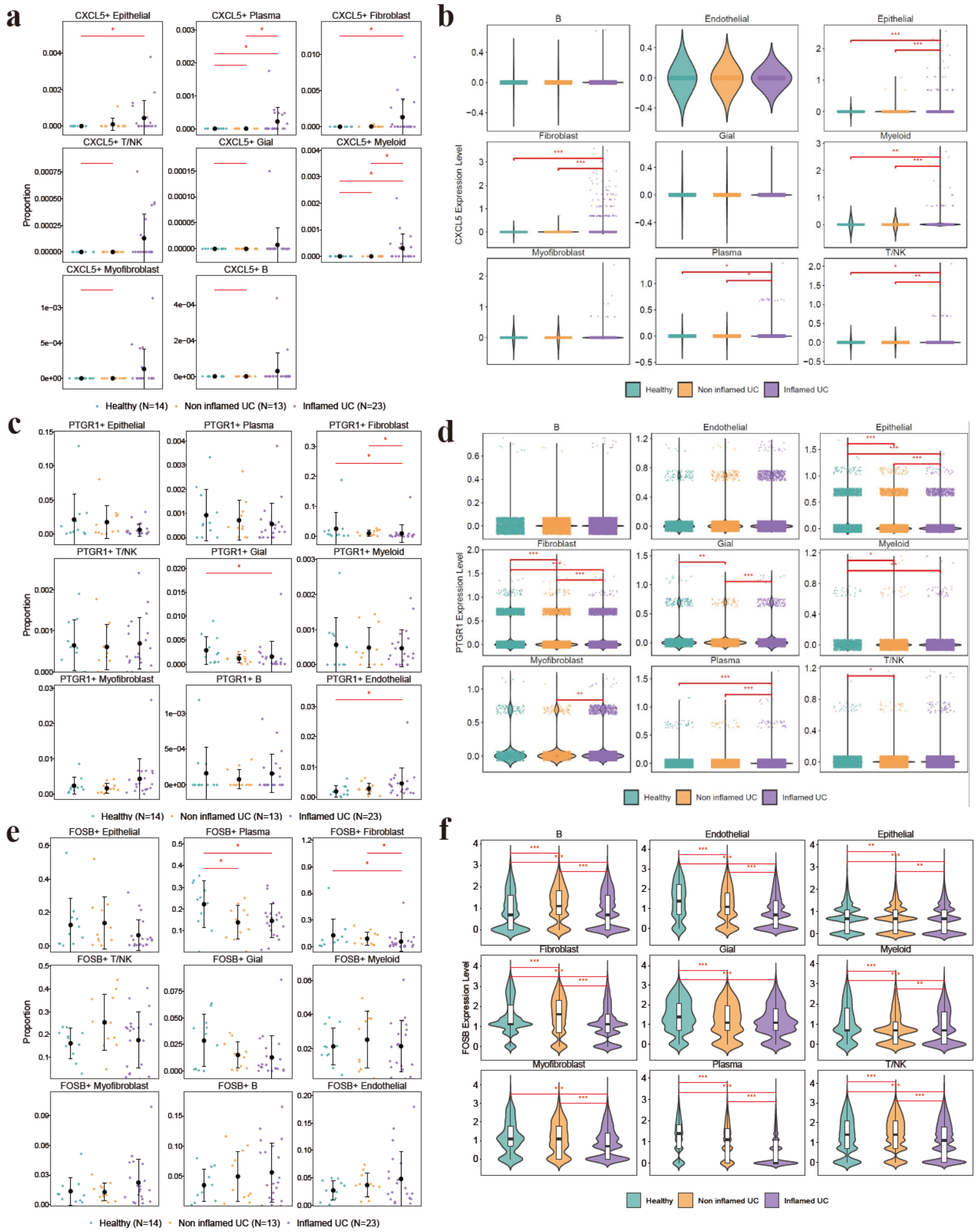

Supplementary Fig. 5. Single-cell transcriptome analysis revealing expression proportion and transcriptomic distribution of the key genes in UC disease progression. a,c,e Scatter plots depicting the proportions of cells expressing marker genes (CXCL5+: **a,** PTGR1+: **c,** FOSB+: **e**) within the specific cell type across healthy control (N=14), non-inflamed UC (N=13), and inflamed UC (N=23) samples, with statistical significance determined by two-sided Wilcoxon rank-sum test (*P<0.05). **b,d,f** Expression of the key genes (CXCL5: **b,** PTGR1: **d,** FOSB: **f**) across annotated cell types in healthy control, non-inflamed UC, and inflamed UC samples, demonstrating differential gene activity between disease states. Statistical significance assessed by two-sided Wilcoxon rank-sum test (*P < 0.05, **P < 0.01, ***P < 0.001).

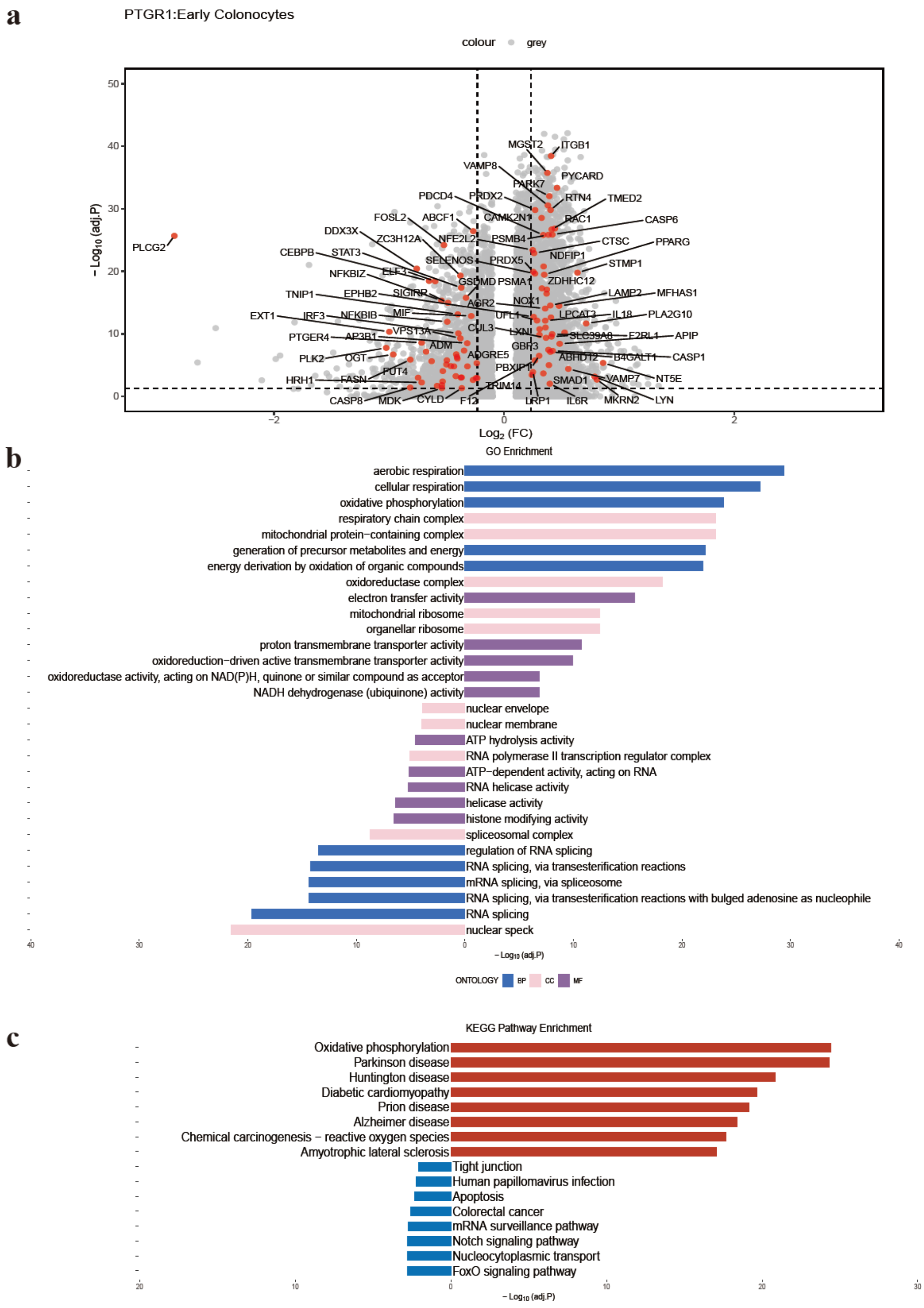

Supplementary Fig. 6. scRNA transcriptomic data profiling reveals PTGR1 associated inflammatory response and pathway dysregulation in UC early colonocytes. a, Volcano plot shows differentially expressed genes (adj.P < 0.05 & $|log_2FC| > 0.232$) in ($PTGR1^+$ vs $PTGR1^-$) early colonocytes. Dashed reference line denotes significance threshold. Red points denote the inflammatory-associated genes (Gene Ontology Term No.0006954: inflammatory response). **b,** GO pathway enrichment plot displays top significantly up-regulated (the right bars) and down-

regulated (the left bars) pathways for the differentially expressed genes of early colonocytes (PTGR1^{+} vs PTGR1^{-}) of UC single-cell datasets. For each category of GO term (BP: biological process; CC: cellular components; MF: molecular function), the top 5 significant terms were selected. **c,** KEGG pathway enrichment plot illustrates top significantly up-regulated (red) and down-regulated (blue) pathways for the differentially expressed genes of early colonocytes (PTGR1^{+} vs PTGR1^{-}) of UC single-cell datasets.

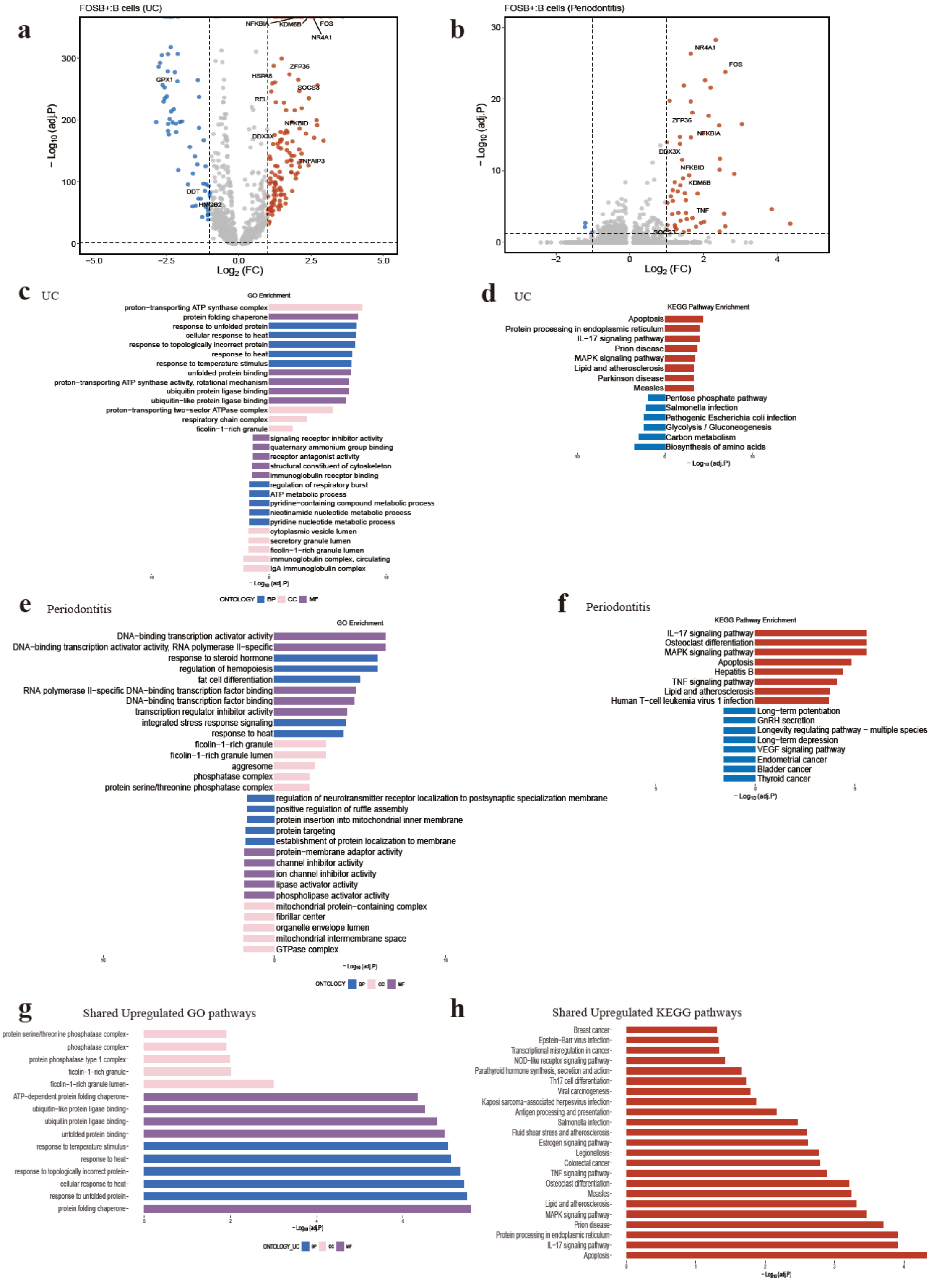

Supplementary Fig. 7. FOSB Expression and Functional Characterization in B Cells in UC and periodontitis. a,b, Volcano plots comparing transcriptional profiles of FOSB$^+$ versus FOSB$^-$ B cells in UC (**a**) and periodontitis (**b**). Differentially expressed genes associated with inflammation activity (Gene Ontology Term No.0006954: inflammatory response) are highlighted. **c,d,** Gene Ontology (GO) (**c**) and KEGG (**d**) pathway enrichment analyses of differentially expressed genes (adj.P < 0.05

& |log2FC| > 1) in UC FOSB+ B cells (FOSB+ B vs. FOSB- B). Left bars: pathways linked to downregulated genes; right bars: pathways linked to upregulated genes. **e,f,** Parallel GO/KEGG enrichment analyses for DEGs in periodontitis $FOSB^{+}$ B cells (FOSB+ B vs. FOSB- B), formatted as in panel **c,d. g,** Common upregulated GO terms enriched in both UC and periodontitis FOSB+ B cells. **h,** Common upregulated KEGG pathway enriched in both UC and periodontitis FOSB+ B cells.

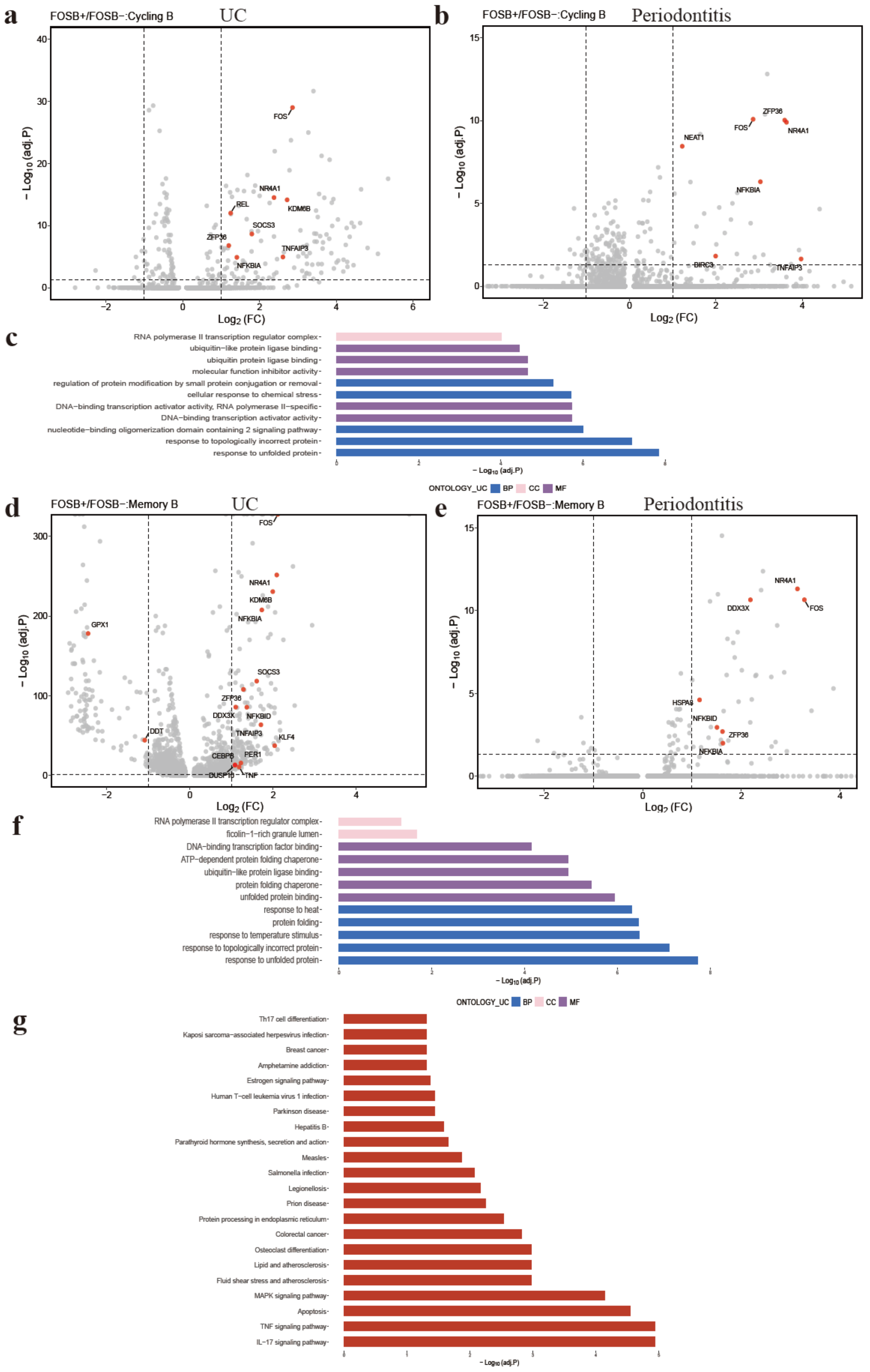

Supplementary Fig. 8. Comparative transcriptional profiling reveals shared inflammatory pathways in FOSB+ B cell subclusters across UC and periodontitis. **a,b,** Volcano plots comparing transcriptional profiles of FOSB^{+} versus FOSB^{-} Cycling

B cells in UC (**a**) and periodontitis (**b**). **c,** The shared significantly upregulated GO pathways enriched in the differentially expressed genes of Cycling B cells (FOSB+ vs. FOSB-) across UC and periodontitis single-cell datasets. **d,e,** Volcano plots comparing transcriptional profiles of $FOSB^{+}$ versus $FOSB^{-}$ Memory B cells in UC (**d**) and periodontitis (**e**). **f,g,** The shared significantly upregulated GO (**f**) and KEGG (**g**) pathways enriched in the differentially expressed genes of Memory B cells (FOSB+ vs. FOSB-) across UC and Periodontitis single-cell datasets.

Differentially expressed genes associated with inflammation activity (Gene Ontology Term No.0006954: inflammatory response) are highlighted. The length of bars denotes the adjusted P values for UC.

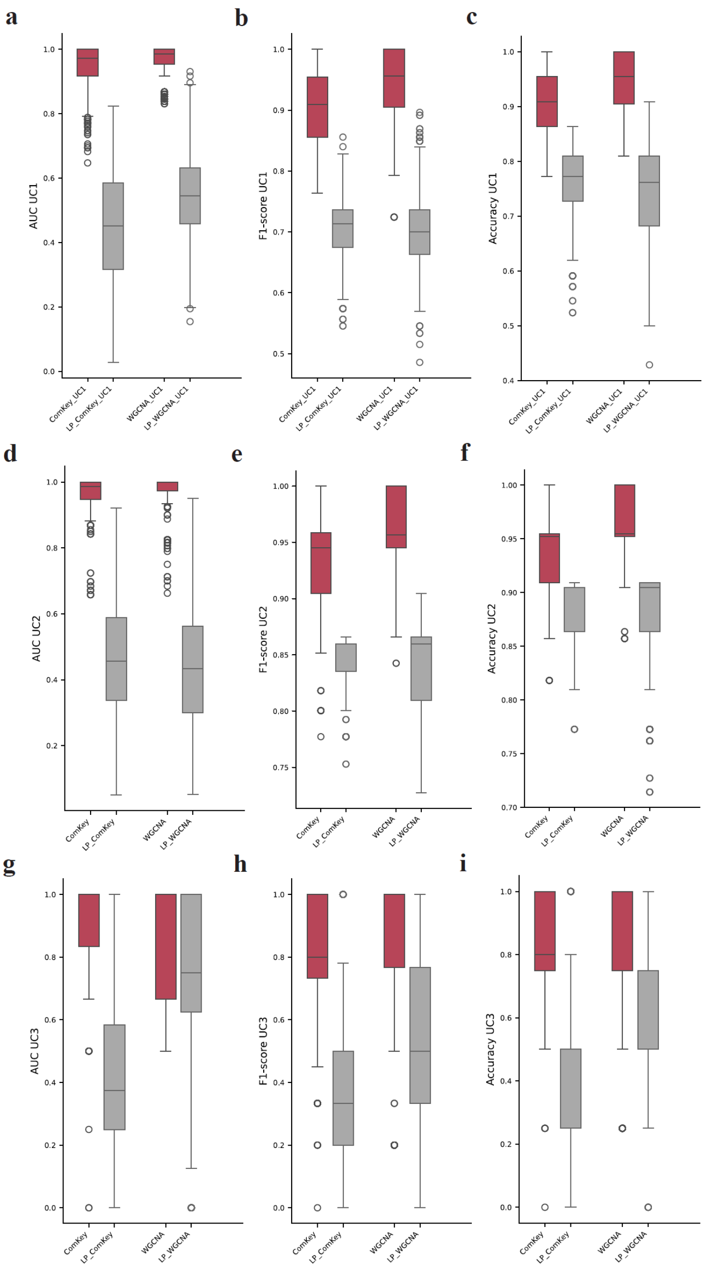

Supplementary Fig. 9. Benchmarking of ComKey and WGCNA-derived gene sets across UC cohorts.

Predictive performance comparison between PMGCN-derived ComKey genes and WGCNA-derived gene sets across three ulcerative colitis cohorts. **a-c,** show AUROC, F1-score, and Accuracy in the UC1 cohort; **d-f,** correspond to the external validation cohort UC2; **g-i,** represent the independent validation cohort UC3. Red boxplots depict the predictive performance of the models based on true labels, and gray boxplots represent performance based on label-permuted samples. Model performance was assessed using nested bootstrapping (200 iterations) with stratified 5-fold cross-validation.

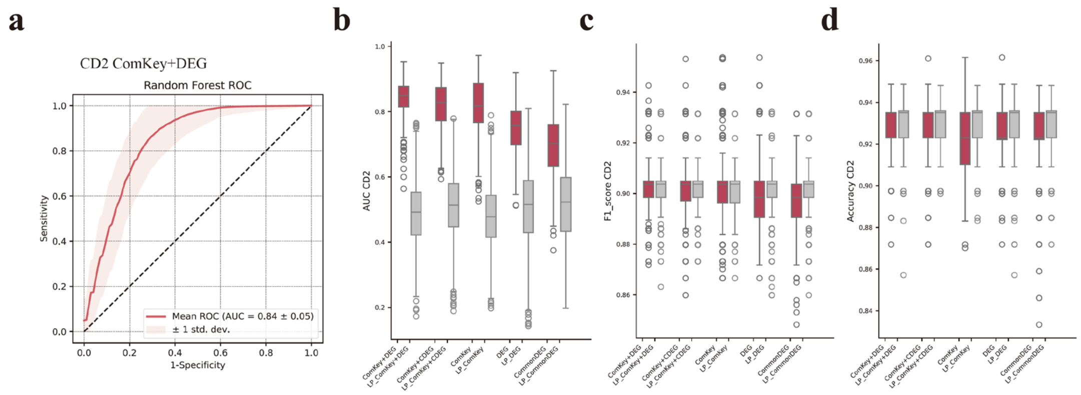

Supplementary Fig. 10. Performance overview of PMGCN for the independent CD2 cohort

Random Forest classification performance evaluated on CD2, an independent external validation dataset. **a,** ROC curve of the ComKey+DEG biomarker panel, showing a mean AUC of 0.84 across bootstrap iterations. **b-d**, Boxplots of AUROC, F1-score, and Accuracy across 200 bootstrapping iterations with stratified 5-fold cross-validation. Red and gray boxplots correspond to performance based on the original and label-permuted data, respectively. All metrics were derived from nested bootstrapping with stratified cross-validation.

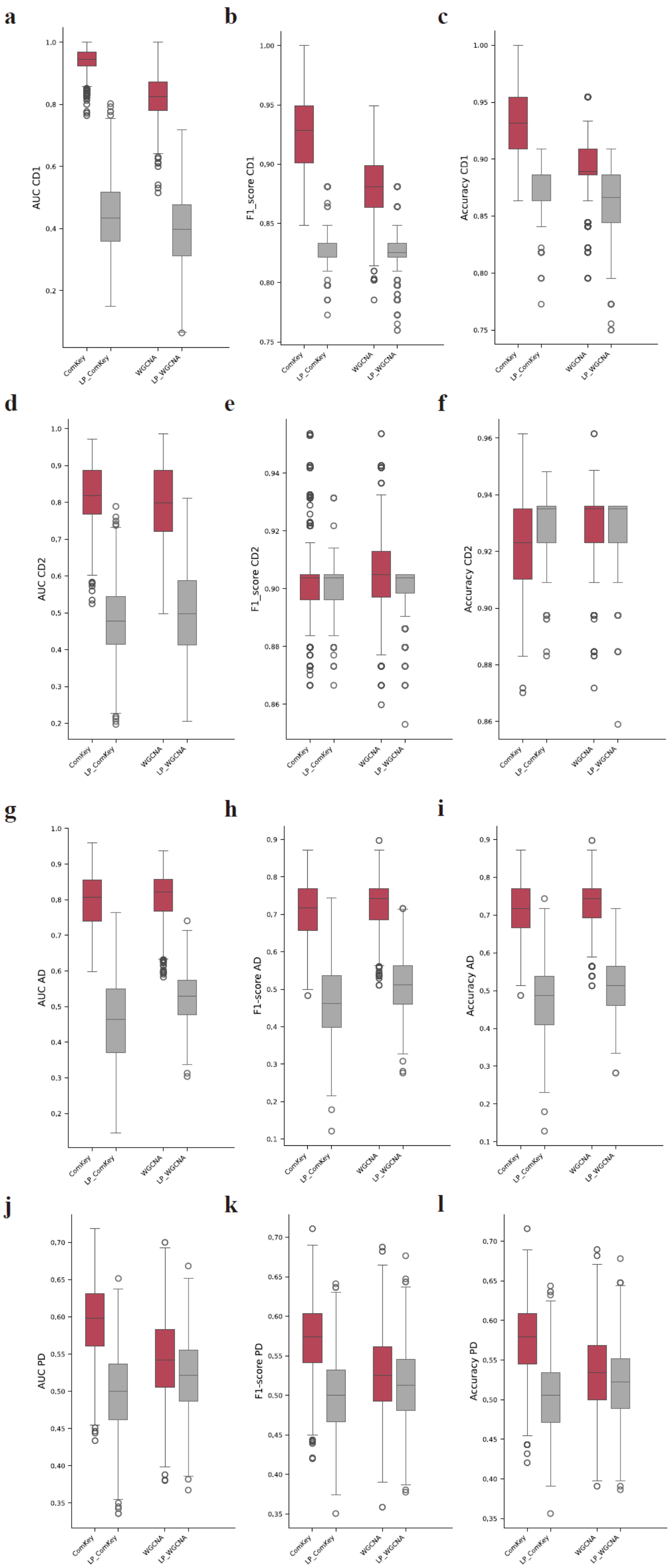
a
AUC CD1
b
F1_score CD1
c
Accuracy CD1
d
AUC CD2
e
F1_score CD2
f
Accuracy CD2
g
AUC AD
h
F1-score AD
i
Accuracy AD
j
AUC PD
k
F1-score PD
l
Accuracy PD
ComKey
LP_ComKey
WGCNA
LP_WGCNA

Supplementary Fig. 11. Benchmarking of ComKey and WGCNA-derived gene sets across CD, UC, and PD cohorts.

Predictive performance comparison between PMGCN-derived ComKey genes and WGCNA-derived gene sets in Crohn's disease (CD), Alzheimer's disease (AD), and Parkinson's disease (PD) cohorts. **a-c,** show AUROC, F1-score, and Accuracy in the CD1 cohort; **d-f,** correspond to the CD2 cohort, an independent external validation dataset; **g-i,** correspond to the AD cohort; **j-l,** correspond to the PD cohort. Red boxplots depict the predictive performance of the models based on true labels, and gray boxplots represent performance based on label-permuted samples. Model performance was assessed using nested bootstrapping (200 iterations) with stratified 5-fold cross-validation.

Supplementary Methods

Weighted Gene Co-expression Network Analysis (WGCNA):

Network Construction

Weighted gene co-expression network analysis was performed using the WGCNA package (*v1.73*) in R (*v4.3.3*) on the processed microarray expression data for each of the four investigated chronic inflammatory disease datasets (UC1, AD, CD1 and PD). The expression matrix was pre-filtered to retain genes with expression values >1 in at least 20% of samples. For computational efficiency, the top 5,000 most variable genes were selected when total genes exceeded this threshold. Sample and gene quality control was performed using the *goodSamplesGenes* function to exclude genes or samples with excessive missing values. Outlier samples were identified and removed via hierarchical clustering of samples with a defined height cutoff (*cutHeight*). A signed weighted adjacency matrix was generated by raising the Pearson correlation matrix to a soft-thresholding power (β), selected based on the scale-free topology criterion. The optimal power was chosen as the smallest value (ranging from 2–20) that achieved a scale-free model fit $R^2 > 0.8$.

Gene Prioritization and Selection

The adjacency matrix of the network was then transformed into a topological overlap matrix (TOM), and a dissimilarity matrix (1 – TOM) was used for hierarchical clustering. Initial co-expression modules were identified using the dynamic tree cut algorithm, with a minimum module size (*minClusterSize*) set. Highly similar modules, based on eigengene correlations, were merged using a predefined merge cutoff (*mergeCutHeight*). Module eigengenes were extracted for each module, and their correlations with the clinical trait were quantified using Pearson correlation coefficients and corresponding p-values. The module with the highest absolute correlation value with clinical trait was identified as the most significant trait-associated module. Hub genes were prioritized based on the product of module membership (*MM*) and the absolute value of gene significance (*GS*) ($MM \times |GS|$). The top *N* genes ranked by this composite score were selected as key genes derived from WGCNA, where *N* corresponded to the number of genes identified for each disease via PMGCN
