# Supplementary material for: Identifying omic biomarkers for chronic inflammatory diseases associated with periodontitis using percolation on multi-disease gene co-expression networks
Source: Commun Med (Lond). 2026 Apr 21;6:351. doi: 10.1038/s43856-026-01591-w (PMC13284180; doi:10.1038/s43856-026-01591-w)
Supplement: Supplementary file 3 — Description of Additional Supplementary files [file 43856_2026_1591_MOESM3_ESM.docx]

**Description of Additional Supplementary Files**

File name: Supplementary Data 1

Description: Optimal percolation algorithms and the identified key genes under the chosen hyperparameters ρ,α

File name: Supplementary Data 2

Description: Shows PMGCN parameter sensitivity analysis across multiple network hyperparameter settings (ρ, α)

File name: Supplementary Data 3

Description: The common DEGs for the periodontitis and UC samples

File name: Supplementary Data 4

Description: The gene compositions of the omic biomarker panels for the four chronic inflammatory diseases

File name: Supplementary Data 5

Description: Source Data behind the figures
