# Supplementary material for: Identifying omic biomarkers for chronic inflammatory diseases associated with periodontitis using percolation on multi-disease gene co-expression networks
Source: Commun Med (Lond). 2026 Apr 21;6:351. doi: 10.1038/s43856-026-01591-w (PMC13284180; doi:10.1038/s43856-026-01591-w)
Supplement: Supplementary file 1 — Transparent Peer Review file [file 43856_2026_1591_MOESM1_ESM.pdf]

nature portfolio

Peer Review File

Identifying omic biomarkers for chronic inflammatory diseases associated with periodontitis using Percolation on Multi-disease Gene Co-expression Networks

Corresponding Author: Dr Shiyong Ma

This file contains all reviewer reports in order by version, followed by all author rebuttals in order by version.

Version 0:

Reviewer comments:

Reviewer #1

(Remarks to the Author)
Major issues:
1) As a methodology article, in the introduction part, the literature reviews only mention the most canonical WGCNA but lack general summaries of past works on the development of multi-disease networks, and co-expression networks. A more specific and relevant computational literature review of the multi-disease network niche is needed to justify the rationale and innovation of the network construction method. In addition, authors have systematically applied the PMGCN method to multiple inflammatory disease; at least on one of the datasets, PMGCN should be compared with one alternative network approach except for DEGs.
2) Neuronal inflammatory disease, such as AD/PD, classification accuracy is a significantly worse than the other inflammatory diseases. Please compare some metrics between pseudobulk expression in single-cell RNA-seq to the bulk microarray data used in the study. This might be caused by heterogeneity of the population or data quality issues. How about applying the method PMGCN to the pseudobulk of the single cell expression to analyze the overlaps of the ComKey with the ones constructed with the microarray?
3) In the abstract and Results Section 1, authors need to clarify PMGCN is designed for bulk microarray data, and the involved single cell data is more about the validation of the discovery of biomarkers.
4) In line 472, the PMGCN constructed network are initializing the nodes from the DEGs, it is not clear to see why ComKey biomarkers expand the DEGs. When calculating the partial correlation, it is also not clear if the correlations are computed on one dataset or multiple datasets, if multiple datasets, what kind of batch correction has been made to the expression?
5) The majority of the results focus on the prioritized ComKey biomarkers: CXCL5, PTGR1, and FOSB,
Minor comment:
1. Please convert text 429-444 and 656-668 to supplementary tables.
2. Processed data is worth depositing to Zenodo for reproducibility purposes.
3. Supplementary tables/figures are not provided.

Reviewer #2

(Remarks to the Author)
Manuscript Overview
In this manuscript the authors present a new approach called Percolation on Multi-disease Gene Co-expression Network (PMGCN). The method is based on the construction of multi-disease gene co-expression networks and combining these in multiplex networks with each layer representing a gene co-expression network for the investigated chronic inflammatory diseases. With emphasis on Periodontitis the authors apply their percolation algorithms were applied to obtain an omics biomarker set for each constructed multi-disease gene co-expression network. Particularly for the Periodontitis-ulcerative colitis (UC) network, they identified CXCL5, FOSB and PTGR1 as the key biomarker set as they were the sequence of genes that caused fragmentation of the largest mutually connected component (LMCC) of the network, into non-extensive disconnected clusters. They further performed functional annotation analysis combined with additional validation using 4 public scRNA-Seq datasets (including trajectory analysis), to show that these biomarkers are linked to altered inflammation signalling pathways in different cell clusters including fibroblasts, epithelial cells and B cells, and are connected to UC and

periodontitis progression.
The authors also performed a Random Forest classification prediction of disease severity and results indicated that their gene biomarkers screened by PMGCN exhibited higher accuracy in predicting several chronic inflammatory diseases, with the exception of UC.

All in all, this is an interesting approach that provides a means of performing “biomarker” detection using network attack methods. However, this manuscript raised a series of concerns and questions and several points were not made very clear in the manuscript (please see below).

Major Comments:
1. How novel is the methodology? Network attacks have been used before. Perhaps not under the banner of “Percolations” however there are notable examples of consecutive node removal methods using co-expression networks in the bibliography ((i) Node interference and robustness: performing virtual knock-out experiments on biological networks: the case of leukocyte integrin activation network. PLoS One. 2014 Feb 20;9(2):e88938. doi: 10.1371/journal.pone.0088938. PMID: 24586448; PMCID: PMC3930642, (ii) Rapid and asymmetric divergence of duplicate genes in the human gene coexpression network. BMC Bioinformatics 7, 46 (2006). https://doi.org/10.1186/1471-2105-7-46, (iii) Co-Expression Network Models Suggest that Stress Increases Tolerance to Mutations) and then using the node interference notion which is based on the general perspective that: “a node is removed from the network and the effects of such removal on the network structure are analyzed”.
2. Why do the authors focus quite extensively on the analysis of periodontitis and UC, while other diseases were utilized only for classification tasks?
3. Why did the authors mention that their methodology is applied on the classification of chronic inflammatory diseases when the method was mostly based on the association between periodontitis and other chronic inflammatory diseases?
4. Why did the authors specifically select a set of number biomarkers? For example, why not use the top 6 common DEGs to compare them with ComKey+CommonDEG or ComKey+DEG?
5. Why did the authors not test the RF model on an external (blind) dataset?
6. Why did the authors follow different steps for the Cell clustering during scRNA-Seq analysis?
7. Although mentioned in the text, a LogFC = 1 is too low as a threshold.
8. The authors mention the microarray data is from different experiments but no batch correction was performed. Perhaps this is necessary, as performed for the scRNA-Seq data (integration and harmonization).
9. Ulcerative colitis (UC), Crohn’s disease (CD), Alzheimer’s disease (AD) and Parkinson’s disease (PD) are used but each time they are compared with periodontitis – Why specifically periodontitis why not compare between all pairs of these diseases.
10. It’s not very compressive whether the 3 biomarkers after the network algorithms are common for periodontitis – UC or whether these 3 were the same for every network pair.
11. No mention of the best strategy (network cores, centrality, etc.,) for each disease network pair. The authors just mention that they picked the best one (i.e., the network contains the least number of nodes before collapsing). Although this information may be in the supplementary files, perhaps a mention in the main text would help make this clearer.
12. Could the strategies be employed when dealing with the integration of these diseases into one network instead of disease network pairs?

Minor Comments
Line 33 hasn’t: please change to has not
Line 55 Crohn’s diseases (CD) and Alzheimer’s diseases (AD): please change to disease
Lines 58- 60 Phrasing needs revising - “Correlation between diseases” is too vague. Which diseases? Correlation of what between them?
Lines 79 -81 “networks encode richer information than the isolated single layer counterparts, multiplex networks are special cases of multi-layer networks where individual nodes are only connected to themselves across layers”: please can you rephrase
Lines 90-94 “We initially constructed the multi-disease gene co-expression networks in the form of multiplex networks with one layer representing gene co-expression of periodontitis and another one for one of the investigated chronic inflammatory diseases, and then optimal percolation algorithms were applied to obtain the parsimonious omic biomarker set for each constructed multi-disease gene co-expression network”: please can you rephrase
Line 119 largest: please change to larger
Line 157 In line with previous, PMGCN genes may serve as a: please delete as
Line 207 periodontists: please change it
Line 214 periodontists: please change it
Line 222 Crohn’s diseases: please change to disease
Lines 286-287 “addition, PTGR1 also presents complementarity with ComDEGs and DEGs, and the samples tend to lie diagonally in the corresponding scatterplots”: please can you rephrase
Lines 384-385 “these genes might present orthogonal functionality and together contribute to improve power for disease prediction”: please can you rephrase

Lines 393-394 “Notably, our study also confirmed that CXCL5 expressed in fibroblasts displays a vital role in connecting with periodontitis and UC”: please can you rephrase
Line 586 “python”: please change to Python
Lines 606-607 “and then only the links, the nodes of whom belong to the same component in all the layers, are reserved”: please can you rephrase
Line 613 “formular”: please change to formula

Lines 636-637 "The effects of performance on the prediction of chronic inflammatory diseases were accessed based on the Random Forest classifications": please can you rephrase
Lines 643-647 "model were calculated, boxplots were generated for visualizations": please can you rephrase
Line 646 "performances": please can you change to 'performance metrics'
Line 646 "(AUROC, F1-Score, Accuracy)": please can you change to 'and Accuracy'
Line 651 "performance": please can you change to 'performance metrics'
Line 652 "Accuracy": please can you change to 'and Accuracy'

Reviewer #3

(Remarks to the Author)
Manuscript Overview
In this manuscript the authors present a new approach called Percolation on Multi-disease Gene Co-expression Network (PMGCN). The method is based on the construction of multi-disease gene co-expression networks and combining these in multiplex networks with each layer representing a gene co-expression network for the investigated chronic inflammatory diseases. With emphasis on Periodontitis the authors apply their percolation algorithms to obtain an omics biomarker set for each constructed multi-disease gene co-expression network. Particularly for the Periodontitis-ulcerative colitis (UC) network, they identified CXCL5, FOSB and PTGR1 as the key biomarker set as they were the sequence of genes that caused fragmentation of the largest mutually connected component (LMCC) of the network, into non-extensive disconnected clusters. They further performed functional annotation analysis combined with additional validation using 4 public scRNA-Seq datasets (including trajectory analysis), to show that these biomarkers are linked to altered inflammation signalling pathways in different cell clusters including fibroblasts, epithelial cells and B cells, and are connected to UC and periodontitis progression.
The authors also performed a Random Forest classification prediction of disease severity and results indicated that their gene biomarkers screened by PMGCN exhibited higher accuracy in predicting several chronic inflammatory diseases, with the exception of UC.

All in all, this is an interesting approach that provides a means of performing "biomarker" detection using network attack methods. However, this manuscript raised a series of concerns and questions and several points were not made very clear in the manuscript (please see below).

Major Comments:
1. How novel is the methodology? Network attacks have been used before. Perhaps not under the banner of "Percolations" however there are notable examples of consecutive node removal methods using co-expression networks in the bibliography ((i) Node interference and robustness: performing virtual knock-out experiments on biological networks: the case of leukocyte integrin activation network. PLoS One. 2014 Feb 20;9(2):e88938. doi: 10.1371/journal.pone.0088938. PMID: 24586448; PMCID: PMC3930642, (ii) Rapid and asymmetric divergence of duplicate genes in the human gene coexpression network. BMC Bioinformatics 7, 46 (2006). https://doi.org/10.1186/1471-2105-7-46, (iii) Co-Expression Network Models Suggest that Stress Increases Tolerance to Mutations) and then using the node interference notion which is based on the general perspective that: "a node is removed from the network and the effects of such removal on the network structure are analyzed".
2. Why do the authors focus quite extensively on the analysis of periodontitis and UC, while other diseases were utilized only for classification tasks?
3. Why did the authors mention that their methodology is applied on the classification of chronic inflammatory diseases when the method was mostly based on the association between periodontitis and other chronic inflammatory diseases?
4. Why did the authors specifically select a set of number biomarkers? For example, why not use the top 6 common DEGs to compare them with ComKey+CommonDEG or ComKey+DEG?
5. Why did the authors not test the RF model on an external (blind) dataset?
6. Why did the authors follow different steps for the Cell clustering during scRNA-Seq analysis?
7. Although mentioned in the text, a LogFC = 1 is too low as a threshold.
8. The authors mention the microarray data is from different experiments but no batch correction was performed. Perhaps this is necessary, as performed for the scRNA-Seq data (integration and harmonization).
9. Ulcerative colitis (UC), Crohn's disease (CD), Alzheimer's disease (AD) and Parkinson's disease (PD) are used but each time they are compared with periodontitis – Why specifically periodontitis why not compare between all pairs of these diseases.
10. It's not very compressive whether the 3 biomarkers after the network algorithms are common for periodontitis – UC or whether these 3 were the same for every network pair.
11. No mention of the best strategy (network cores, centrality, etc.,) for each disease network pair. The authors just mention that they picked the best one (i.e., the network contains the least number of nodes before collapsing). Although this information may be in the supplementary files, perhaps a mention in the main text would help make this clearer.
12. Could the strategies be employed when dealing with the integration of these diseases into one network instead of disease network pairs?

Minor Comments
Line 33 hasn't: please change to has not
Line 55 Crohn's diseases (CD) and Alzheimer's diseases (AD): please change to disease
Lines 58- 60 Phrasing needs revising - "Correlation between diseases" is too vague. Which diseases? Correlation of what

between them?
Lines 79 -81 “networks encode richer information than the isolated single layer counterparts, multiplex networks are special cases of multi-layer networks where individual nodes are only connected to themselves across layers”: please can you rephrase
Lines 90-94 “We initially constructed the multi-disease gene co-expression networks in the form of multiplex networks with one layer representing gene co-expression of periodontitis and another one for one of the investigated chronic inflammatory diseases, and then optimal percolation algorithms were applied to obtain the parsimonious omic biomarker set for each constructed multi-disease gene co-expression network”: please can you rephrase
Line 119 largest: please change to larger
Line 157 In line with previous, PMGCN genes may serve as a: please delete as
Line 207 periodontists: please change it
Line 214 periodontists: please change it
Line 222 Crohn’s diseases: please change to disease
Lines 286-287 “addition, PTGR1 also presents complementarity with ComDEGs and DEGs, and the samples tend to lie diagonally in the corresponding scatterplots”: please can you rephrase
Lines 384-385 “these genes might present orthogonal functionality and together contribute to improve power for disease prediction”: please can you rephrase

Lines 393-394 “Notably, our study also confirmed that CXCL5 expressed in fibroblasts displays a vital role in connecting with periodontitis and UC”: please can you rephrase
Line 586 “python”: please change to Python
Lines 606-607 “and then only the links, the nodes of whom belong to the same component in all the layers, are reserved”: please can you rephrase
Line 613 “formular”: please change to formula
Lines 636-637 “The effects of performance on the prediction of chronic inflammatory diseases were accessed based on the Random Forest classifications”: please can you rephrase
Lines 643-647 “model were calculated, boxplots were generated for visualizations”: please can you rephrase
Line 646 “performances”: please can you change to ‘performance metrics’
Line 646 “(AUROC, F1-Score, Accuracy)”: please can you change to ‘and Accuracy’
Line 651 “performance”: please can you change to ‘performance metrics’
Line 652 “Accuracy”: please can you change to ‘and Accuracy’

Version 1:

Reviewer comments:

Reviewer #1

(Remarks to the Author)
Here are my feedbacks to their new version of manuscript:

Overall, the manuscript has been largely improved after addressing all of our questions. This manuscript is more likely to be a biological analysis scope instead of a generalized method since in terms of the network construction goal, this has been attempted by many generic network methods such as the one mentioned WGCNA and CellNet that are developed and benchmarked in more contexts.
As a methodology, the software is not well-maintained as a ready-to-go package and is not comprehensively benchmarked under more association of diseases, I wonder how many network methods are really addressing this unique angle. I again strongly recommended uploading the data to somewhere open-source platform and make the codebase more distributable and installable.
On the other hand, the uniqueness of this study is addressing inflammatory disease association with periodontitis in a conceptually different ways: percolation with multi-layer networks representation. Authors may need to highlight their efforts in this angle of biological discoveries instead of comparing to the generic network method, e.g. how robustness is their discoveries of the three markers and their association with the percolation, this may need some validation. They also need to highlight the periodontitis association in the title.

Reviewer #2

(Remarks to the Author)
The authors have addressed all of the issues raised.

Reviewer #3

(Remarks to the Author)
I co-reviewed this manuscript with one of the reviewers who provided the listed reports. This is part of the Communications Medicine initiative to facilitate training in peer review and to provide appropriate recognition for Early Career Researchers who co-review manuscripts.

Open Access This Peer Review File is licensed under a Creative Commons Attribution 4.0 International License, which permits use, sharing, adaptation, distribution and reproduction in any medium or format, as long as you give appropriate credit to the original author(s) and the source, provide a link to the Creative Commons license, and indicate if changes were made.
In cases where reviewers are anonymous, credit should be given to 'Anonymous Referee' and the source.
The images or other third party material in this Peer Review File are included in the article's Creative Commons license, unless indicated otherwise in a credit line to the material. If material is not included in the article's Creative Commons license and your intended use is not permitted by statutory regulation or exceeds the permitted use, you will need to obtain permission directly from the copyright holder.
To view a copy of this license, visit https://creativecommons.org/licenses/by/4.0/

Referee expertise:

Referee #1: Immunology, inflammatory diseases, biomarker, gene expression, single-cell genomics

Referee #2: Bioinformatics, computational biology, omics framework, RNA biology, inflammatory disease

Referee #3: ECR co-reviewed the paper with one of the reviewers

Reviewers' comments:

Reviewer #1 (Remarks to the Author):

Major issues:

1) As a methodology article, in the introduction part, the literature reviews only mention the most canonical WGCNA but lack general summaries of past works on the development of multi-disease networks, and co-expression networks. A more specific and relevant computational literature review of the multi-disease network niche is needed to justify the rationale and innovation of the network construction method. In addition, authors have systematically applied the PMGCN method to multiple inflammatory disease; at least on one of the datasets, PMGCN should be compared with one alternative network approach except for DEGs.

Response: We thank the reviewer for this critical and insightful comment. We have substantially revised the introduction and conducted new benchmarking analyses to address these points.

Part 1: Comprehensive Literature Review and Methodological Rationale

In the revised introduction, we review recent computational frameworks in network biology specifically designed for multi-disease analysis. These methods are organized by the hierarchical levels at which they compare network differences. We then articulate the specific gap that PMGCN aims to fill.

Part 2: Benchmarking Against an Alternative Network Approach

Following the reviewer's suggestion, we have performed an additional comparative analysis, benchmarking PMGCN against one of the most canonical network methods: Weighted Gene Co-expression Network Analysis (WGCNA).

The corresponding methods (see Supplementary Methods) and results have been added to the revised manuscript, with performance evaluations shown in Supplementary Fig. 9 and Supplementary Fig. 11.

2) Neuronal inflammatory disease, such as AD/PD, classification accuracy is a significantly worse than the other inflammatory diseases. Please compare some metrics between pseudobulk expression in single-cell RNA-seq to the bulk microarray data used in the study. This might be caused by heterogeneity of the population or data quality issues. How about applying the method PMGCN to the pseudobulk of the single cell expression to analyze the overlaps of the ComKey with the ones constructed with the microarray?

Response: We thank the reviewer for this insightful comment and suggestion.

Following the reviewer's suggestion, we directly compared the heterogeneity of the molecular landscapes captured by the two technologies: We assessed transcriptional heterogeneity in Parkinson's disease (PD) by comparing single-nucleus RNA-seq (snRNA-seq)–derived pseudobulk expression profiles with bulk microarray data. The snRNA-seq dataset (GSE202210) was generated from postmortem prefrontal cortex samples of 6 late-stage PD patients and 6 age-matched healthy controls. Pseudobulk expression profiles were constructed by summing raw counts of all single cell within each sample. Subsequently, both pseudobulk and bulk microarray data were processed using an identical pipeline, including mapping to HGNC gene symbols to remove non-standard genes and log2 transformation.

Transcriptional heterogeneity was quantified using the Ratio of Global Unshifted Entropy (ROGUE)[1]. ROGUE scores were calculated under multiple clustering scales (K = 1, 5, 10, 20, 45, 100, 200, and 500), and the trends of ROGUE values across different K values were compared between pseudobulk and bulk datasets (Figure below).

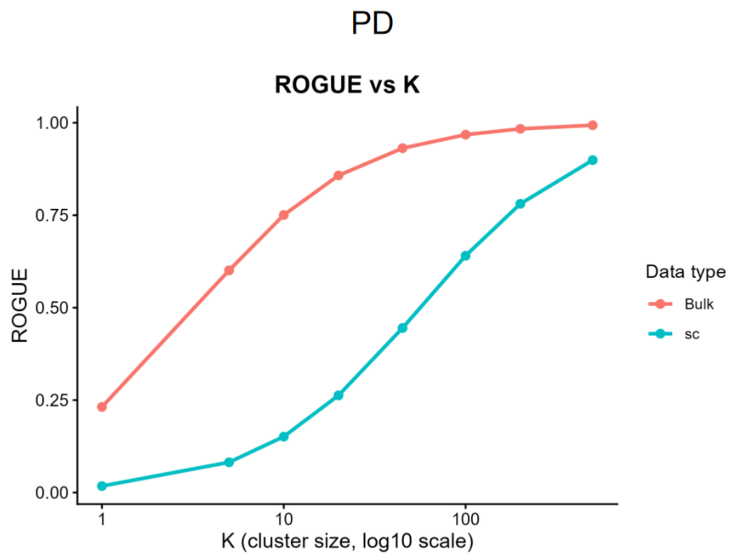

ROGUE analysis revealed that, across all tested K values, snRNA-seq pseudobulk data consistently exhibited lower ROGUE scores than bulk microarray data, indicating a higher level of transcriptional heterogeneity retained in the pseudobulk profiles. Notably, even at large K values, pseudobulk data remained less homogeneous than bulk data, suggesting that PD samples exhibit complex cellular composition and transcriptional state heterogeneity.

Moreover, we actively sought to apply PMGCN to a pseudobulk representation of a large-scale AD/PD single-cell dataset. However, we encountered a significant practical constraint: to our knowledge, currently available single-cell RNA-seq datasets for AD/PD that are suitable for robust pseudobulk analysis (i.e., containing a sufficient number of individual samples per condition to reliably reconstruct population-level networks) are limited. The datasets we found are often structured as few samples with a high cell count per sample, which is insufficient for building the sample-cohort-level correlation networks required by the PMGCN pipeline. Therefore, applying PMGCN directly to pseudobulk data would likely require methodological adaptation. We look forward to optimizing PMGCN framework to suitable, large-scale single-cell datasets in the future.

3) In the abstract and Results Section 1, authors need to clarify PMGCN is designed for bulk microarray data, and the involved single cell data is more about the validation of the discovery of biomarkers.

Response: We sincerely thank the reviewer for this helpful comment.

In response to this comment, we have revised the manuscript to eliminate the ambiguity. The specific changes are as follows:
In Abstract, a statement “derived from bulk transcriptomic gene expression profiles” were added. We have added a clarifying statement “constructed from bulk transcriptomic profiles” in Results Section 1.

4) In line 472, the PMGCN constructed network are initializing the nodes from the DEGs, it is not clear to see why ComKey biomarkers expand the DEGs. When calculating the partial correlation, it is also not clear if the correlations are computed on one dataset or multiple datasets, if multiple datasets, what kind of batch correction has been made to the expression?

Response: We apologize for the lack of clarity and thank the reviewer for allowing us to clarify these key methodological points.

Why ComKey Biomarkers Expand Beyond DEGs:
The PMGCN framework is intentionally designed to start from a broader set of genes than those identified by traditional, stringent DEG criteria and thresholds. We first selected an initial gene set using a relaxed threshold: the threshold for adjusted P

value was determined as 0.05, however, for the threshold with regard to LogFC, we didn't choose a fixed value such as 1, and applied relaxed thresholds (the top ρ percent) which turns out to be substantially smaller than 1. The aim is to ensure that an appropriate number of genes are selected for network construction, and this captures genes with more subtle but potentially coordinated expression changes. This relaxed DEG set serves as the initial nodes for constructing the network. PMGCN can highlight ComKey genes that, while only passing the relaxed DEG threshold, serve crucial functions for the multi-disease analysis. We have further clarified this point in the Methods section 4 (Construction of the Multi-Disease Gene Co-expression Networks) of the revised manuscript.

Partial Correlation Calculation and Batch Correction:
The partial correlation network was computed on single dataset for each chronic inflammatory disease in this study. Since the analysis was performed on single batch of data, there was no requirement for batch correction in this specific step. We have clarified this point in the Methods section 1 (Setting up bulk gene-expression dataset) of the revised manuscript to prevent this misunderstanding.

5) The majority of the results focus on the prioritized ComKey biomarkers: CXCL5, PTGR1, and FOSB,

Response: We thank the reviewer for raising this important point. Our selection was not arbitrary but was designed to provide a detailed, mechanistic case study to concretely illustrate the discovery power of the PMGCN framework. For instance, while FOSB might not rank among the top DEG by traditional analysis, its identification as a top ComKey biomarker by PMGCN suggests it plays a critical role in the UC-Periodontitits network, a finding that would be missed by standard DEG analysis alone. To provide a more comprehensive view of all findings, the complete list of ComKey biomarkers identified for each inflammatory disease were provided in Supplementary Data 1.

Minor comment:

1. Please convert text 429-444 and 656-668 to supplementary tables.

Response: We have converted the text in lines 429-444 (which describes the collected bulk microarray datasets) into Supplementary Table 1, and the text in lines 656-668 (which describes the collected single-cell RNA-Seq datasets) into Supplementary Table 2. The main text has been revised to refer to these new supplementary tables.

2. Processed data is worth depositing to Zenodo for reproducibility purposes.

Response: We thank the reviewer for this suggestion regarding data deposition. In our

study, the microarray and single-cell RNA-seq data were obtained directly from public repositories (via the *GEOquery* package) and analyzed using a series of R packages. To ensure full reproducibility, we have deposited the complete analysis code on GitHub (link provided in the manuscript), which allows any user to download the raw data from the original sources and reproduce all processing steps and results. Given that the data are publicly accessible and our code fully documents the analysis pipeline, we believe that depositing the processed data on Zenodo is not essential for reproducibility. However, if there are specific processed datasets or intermediate files that the reviewer would find valuable for further exploration, please let us know - we would be happy to upload those files to Zenodo or another appropriate repository.

3. Supplementary tables/figures are not provided.

Response: We sincerely apologize for any confusion. We have double-checked that the file has been successfully uploaded with the revised manuscript.

Reviewer #2 (Remarks to the Author):

Manuscript Overview

In this manuscript the authors present a new approach called Percolation on Multi-disease Gene Co-expression Network (PMGCN). The method is based on the construction of multi-disease gene co-expression networks and combining these in multiplex networks with each layer representing a gene co-expression network for the investigated chronic inflammatory diseases. With emphasis on Periodontitis the authors apply their percolation algorithms were applied to obtain an omics biomarker set for each constructed multi-disease gene co-expression network. Particularly for the Periodontitis-ulcerative colitis (UC) network, they identified CXCL5, FOSB and PTGR1 as the key biomarker set as they were the sequence of genes that caused fragmentation of the largest mutually connected component (LMCC) of the network, into non-extensive disconnected clusters. They further performed functional annotation analysis combined with additional validation using 4 public scRNA-Seq datasets (including trajectory analysis), to show that these biomarkers are linked to altered inflammation signalling pathways in different cell clusters including fibroblasts, epithelial cells and B cells, and are connected to UC and periodontitis progression.

The authors also performed a Random Forest classification prediction of disease severity and results indicated that their gene biomarkers screened by PMGCN exhibited higher accuracy in predicting several chronic inflammatory diseases, with the exception of UC.

All in all, this is an interesting approach that provides a means of performing "biomarker" detection using network attack methods. However, this manuscript raised a series of concerns and questions and several points were not made very clear in the manuscript (please see below).

Major Comments:
1. How novel is the methodology? Network attacks have been used before. Perhaps not under the banner of "Percolations" however there are notable examples of consecutive node removal methods using co-expression networks in the bibliography ((i) Node interference and robustness: performing virtual knock-out experiments on biological networks: the case of leukocyte integrin activation network. PLoS One. 2014 Feb 20;9(2):e88938. doi: 10.1371/journal.pone.0088938. PMID: 24586448; PMCID: PMC3930642, (ii) Rapid and asymmetric divergence of duplicate genes in the human gene coexpression network. BMC Bioinformatics 7, 46 (2006). https://doi.org/10.1186/1471-2105-7-46, (iii) Co-Expression Network Models Suggest that Stress Increases Tolerance to Mutations) and then using the node interference notion which is based on the general perspective that: "a node is removed from the network and the effects of such removal on the network structure are analyzed".

Response: We thank the reviewer for raising this important point regarding methodological novelty. We agree that the concept of "node removal" or "network attack" is well-established in network science. However, the core novelty of our PMGCN approach lies not in the concept of node removal itself, but in its *application within a specifically constructed multiplex gene co-expression network framework* and the use of *optimal percolation theory* to identify a minimal biomarker set.

Key Differentiators:
Multiplex Network Framework: Unlike the cited studies which perform attacks on single-layer networks, PMGCN operates on a multiplex network where two disease-specific gene co-expression networks (e.g., periodontitis and UC) are interconnected. This structure allows us to model the shared genetic architecture between two diseases explicitly. The percolation attack is performed on this interconnected system, targeting the *Largest Mutually Connected Component (LMCC)*, which represents genes that are co-expressed and functionally coherent in *both* diseases.
Objective: Our goal is not just to attack the network or analyze the effect of node removal, but to find the *minimum sequence* of gene removals that causes the collapse of the LMCC. This "optimal percolation" approach is theoretically grounded in identifying the most influential nodes for global connectivity, which we repurpose to

find a parsimonious, high-impact biomarker set.
Cross-Disease Biomarker Discovery: The primary application is the identification of biomarkers that are critically important for the shared pathology of two distinct but related chronic inflammatory diseases, which, to our knowledge, has not been done using this specific combination of multiplex networks and optimal percolation.

In response to this comment, we have revised the manuscript to clarify the point and have added a discussion citing these references in the Discussion section.

2. Why do the authors focus quite extensively on the analysis of periodontitis and UC, while other diseases were utilized only for classification tasks?

Response: Thanks for the reviewer's excellent question.

We extensively focused on ulcerative colitis (UC) and periodontitis for several reasons. First, periodontitis has been widely reported to be significantly associated with UC, with substantial research supporting their correlation. The link between oral and intestinal diseases has become a research focus in recent years [2,3]. Second, through searches in public databases, we were able to obtain abundant bulk omics and single-cell RNA sequencing data, which provide a solid foundation for analytical validation and algorithm verification.

Therefore, we selected the Periodontitis-UC pair as our primary, in-depth case study to validate the biological plausibility of the PMGCN-derived biomarkers through extensive functional annotation and scRNA-seq analysis. The other disease pairs (Periodontitis-CD, -AD, -PD) served as validation cohorts to demonstrate the generalizability of the PMGCN method itself. Showing that the same computational pipeline could generate effective biomarker sets for different disease pairs strengthens the claim that PMGCN is a generally applicable tool, not just a one-off finding for Periodontitis-UC. We have clarified this rationale in the introduction section of the revised manuscript.

3. Why did the authors mention that their methodology is applied on the classification of chronic inflammatory diseases when the method was mostly based on the association between periodontitis and other chronic inflammatory diseases?

Response: Thanks for this insightful comment. Since Questions 3 and 9 are interrelated, we address them together in this combined reply. The reviewer rightly points out that our network construction was centered on pairwise associations with periodontitis. We selected periodontitis as the fixed disease for the following methodological and biological reasons:

1) **Methodological Focus & Simplification:** As a methodological study, our primary aim was to establish a clear proof-of-concept for the PMGCN framework. Pairwise comparisons across all possible chronic inflammatory diseases would introduce excessive complexity at this stage. Using periodontitis as a fixed node provides a simplified yet biologically meaningful scaffold to validate the model's core functionality.
2) **Strong Biological Rationale:** Periodontitis is a well-established systemic inflammatory trigger and a known risk factor for several chronic inflammatory diseases. Its specific links via the "oral-gut axis" (with UC/CD) and "oral-brain axis" (with AD/PD) provided a biologically grounded use case for evaluating our approach.

To directly address the question of generalizability beyond periodontitis-centric pairs, we applied PMGCN to other chronic inflammatory disease pairs. The results confirm the method's broader applicability:

- **CD-UC**: Identification of seven ComKey genes (CLDN18, MMP7, CCL20, CR2, IL1A, SDR16C5, and NR4A2) yielded an AUC of 0.94 in the original UC (UC1) dataset (Figure below), and 0.96/0.95 in two external UC datasets (UC2/UC3), while for CD the AUC was 0.78 (CD1) and 0.66 (CD2, external).

UC1

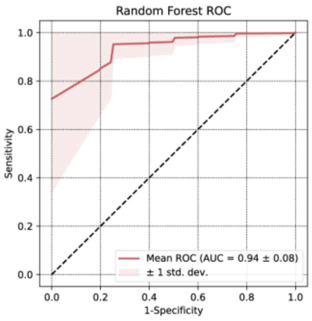

UC2

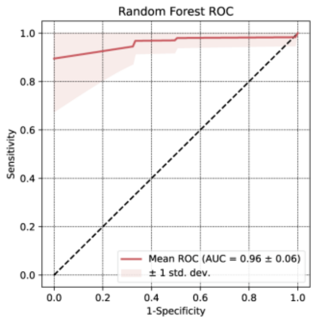

UC3

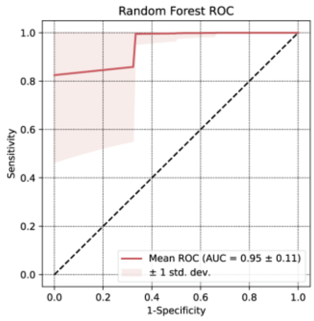

CD1

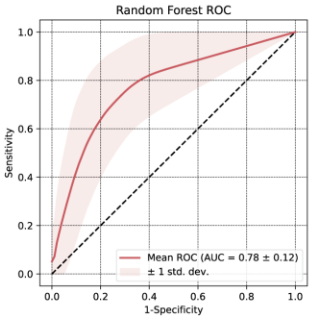

CD2

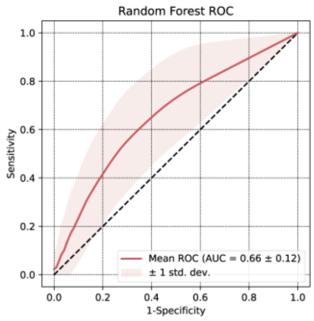

- **AD-UC:** Three ComKey genes (SRD5A3, MT1G, RGS1) were identified, with performance detailed in the figure below.

UC1

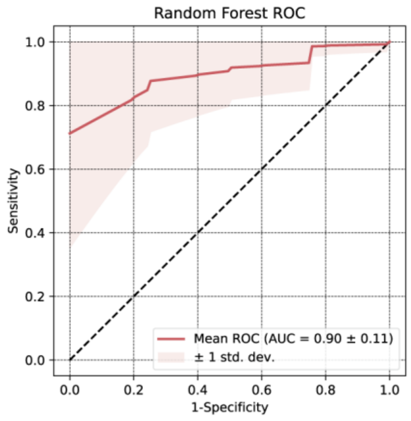

UC2

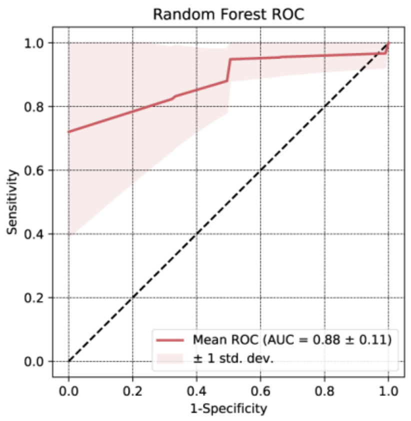

UC3

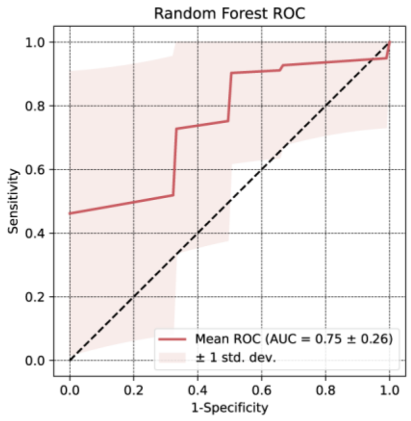

AD

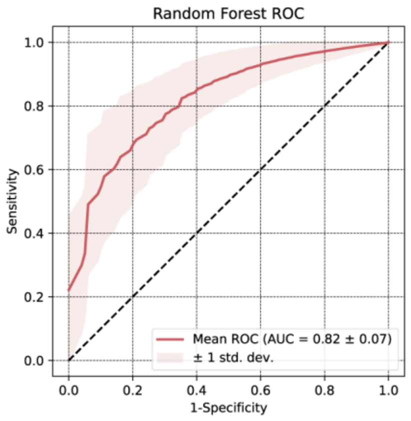

- **AD-PD:** Four ComKey genes (CYP4F2, CHST2, XHPR, and URB1) were identified, with results shown in the figure below.

AD

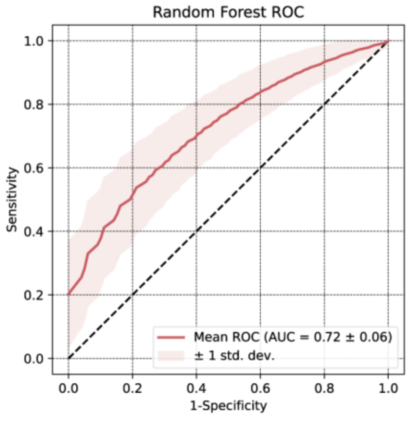

PD

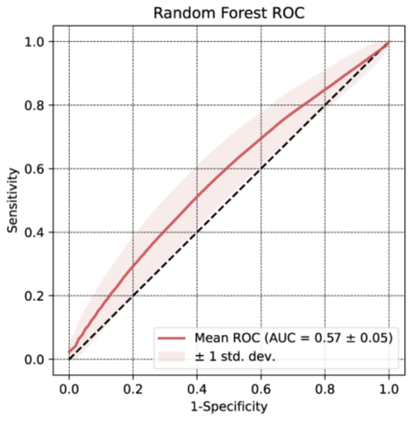

These outcomes confirm that although PMGCN performs most strongly in the periodontitis-UC context - likely reflecting their pronounced biological linkage - it remains capable of identifying predictive genes in other disease pairs, supporting its

broader applicability across chronic inflammatory conditions. We have incorporated a discussion of this point in the revised manuscript to ensure clarity.

4. Why did the authors specifically select a set of number biomarkers? For example, why not use the top 6 common DEGs to compare them with ComKey+CommonDEG or ComKey+DEG?

Response: The number of biomarkers (e.g., the final 3 for Periodontitis-UC) was not arbitrarily chosen by us. It was determined by the percolation algorithm. The algorithm sequentially removes genes that cause the largest drop in LMCC size. The point at which the network fragments into non-extensive clusters defines the minimal critical set. In the case of Periodontitis-UC, this set consisted of CXCL5, FOSB, and PTGR1. The number of biomarkers used for comparison is determined by the size of ComKey (n=3).

5. Why did the authors not test the RF model on an external (blind) dataset?

Response: Thanks for the reviewer's comment. In the manuscript, we have sourced and incorporated two independent, publicly available microarray datasets for Ulcerative Colitis (UC2: GSE59071; UC3: GSE48958) that was not used in any phase of PMGCN for biomarker discovery. We used the identified PMGCN biomarkers (CXCL5, FOSB, PTGR1) to predict disease state (UC vs. Control) in these new datasets using the RF model. The results show promising discriminatory power (AUROC > 0.75), providing independent validation of our biomarkers. In our revised manuscript, we have undertaken additional effort to further address this. An external CD dataset (CD2: GSE112366) was included, showing that ComKey has an AUC of 0.77, CommonDEG 0.65, and DEG 0.70. This new analysis has been added to the results section (PMGCN improves predictability for other chronic inflammatory diseases) and results are shown in Supplementary Fig. 10. For AD and PD, we did not find suitable datasets.

6. Why did the authors follow different steps for the Cell clustering during scRNA-Seq analysis?

Response: We apologize for the lack of clarity. The underlying clustering principles were consistent (graph-based clustering on PCA-reduced data from highly variable genes). The differences in the described steps (e.g., resolution parameters, specific genes used for annotation) were tailored to the individual datasets because they originated from different studies, had different cell type compositions, and required dataset-specific fine-tuning to achieve biologically meaningful clusters. We have revised the methods section (scRNA-Seq data analysis) to clarify this point.

7. Although mentioned in the text, a LogFC = 1 is too low as a threshold.

Response: Thanks for the reviewer's insightful comment regarding the threshold (|log2FC| > 1) used for identifying differentially expressed genes (DEGs). We appreciate the opportunity to further clarify our methodological choice.

The primary objective of our conventional DEG analysis (applied to both bulk and single-cell omics data) was to provide an initial, broad characterization of biological pathways potentially involved in the pathogenesis of chronic inflammatory disease—ulcerative colitis (UC). This approach is intentionally exploratory. We adopted a relatively inclusive threshold ($|\log_2 FC| > 1$) to capture a richer set of genes for downstream pathway enrichment analysis, thereby allowing a more comprehensive preliminary survey of mechanistic landscapes. Our focus was not on applying stringent criteria to identify statistically stable biomarkers at this stage, but on generating hypotheses for subsequent interpretation.

We also note that a threshold of $|\log_2 FC| > 1$ is not universally considered low. For instance, in single-cell RNA-seq studies - where data sparsity and technical noise are prominent - even lower thresholds (e.g., $|\log_2 FC| = 0.3–0.5$) are sometimes employed in well-regarded research [4].

In summary, our threshold selection was driven by the exploratory nature of this specific analysis phase within our study. We believe it was appropriate for achieving the stated goal of pathway-oriented discovery and has yielded biologically meaningful insights, as detailed in the manuscript. We are happy to discuss this further or to provide additional clarification if needed.

8. The authors mention the microarray data is from different experiments but no batch correction was performed. Perhaps this is necessary, as performed for the scRNA-Seq data (integration and harmonization).

Response: Thanks for the reviewer's comment regarding batch effects. In our study, although we gathered multiple microarray datasets for some diseases, our analytical strategy for constructing gene co-expression networks did not involve pooling datasets together for a single, integrated analysis. Instead, for each disease, we selected the dataset with the largest sample size or the most balanced sample composition to build and analyze the co-expression network. The primary role of the remaining datasets was to serve as independent validation cohorts, specifically used to assess the robustness and predictive performance of the biomarkers. Therefore, we believe that batch effect correction was not a necessary step for the analytical pipeline of our study.

9. Ulcerative colitis (UC), Crohn's disease (CD), Alzheimer's disease

(AD) and Parkinson' s disease (PD) are used but each time they are compared with periodontitis – Why specifically periodontitis why not compare between all pairs of these diseases.

Response: This point has been addressed in our response to Question 3.

10. It' s not very compressive whether the 3 biomarkers after the network algorithms are common for periodontitis – UC or whether these 3 were the same for every network pair.

Response: We apologize for the lack of clarity. The three biomarkers (CXCL5, FOSB, PTGR1) are specific to the Periodontitis-UC network pair. Each disease pair (e.g., Periodontitis-CD, Periodontitis-AD) yielded a unique, distinct set of key biomarkers through the PMGCN process, as the underlying co-expression networks and their interconnectivity are different. We have created Supplementary Data 1 that lists the key biomarker sets for all four disease pairs investigated and have emphasized this in the results section (PMGCN improves predictability for other chronic inflammatory diseases) to avoid any confusion.

11. No mention of the best strategy (network cores, centrality, etc.,) for each disease network pair. The authors just mention that they picked the best one (i.e., the network contains the least number of nodes before collapsing). Although this information may be in the supplementary files, perhaps a mention in the main text would help make this clearer.

Response: We thank the reviewer for this valuable suggestion. In Results section 1, we explicitly stated that for the Periodontitis-UC disease group, the CoreHD and DCI algorithms with Pareto optimality were used due to the minimal node set obtained. In Results section 7, we have added descriptions indicating the optimal algorithm identified for each of the remaining disease pairs. We hope these revisions ensure improved transparency.

12. Could the strategies be employed when dealing with the integration of these diseases into one network instead of disease network pairs?

Response: Thanks for the reviewer's suggestion. This is a fascinating and ambitious idea. Yes, the PMGCN framework could theoretically be extended to a multi-layer network with more than two layers (e.g., one layer each for Periodontitis, UC, CD, AD, PD). The definitions of mutual connectivity and the percolation algorithms would be rescaled accordingly.

Following the reviewer's comment, we explored integrating all five diseases into a

single multiplex network. Briefly, for each disease, genes with adjusted p-value < 0.05 (without FC restriction) were selected; the intersection across all five diseases yielded 136 genes. Co-expression networks were constructed per disease, retaining edges with probability > 0.9, and the networks were merged using periodontitis nodes as reference, resulting in a union network with 84 nodes and 66 edges. Applying PMGCN on this union network identified three ComKey genes (KRT23, CFP, and TEAD2). Prediction performance (Random Forest) was reasonable (UC AUC 0.89, external UC 0.86/0.79; CD 0.83, external 0.53; AD 0.83; PD 0.56), the figures are given below. The overall performance was lower than the pairwise disease analyses. This exploratory analysis demonstrates the feasibility of a fully integrated multi-disease network, though further optimization is needed, and we plan to investigate this in future work.

UC1

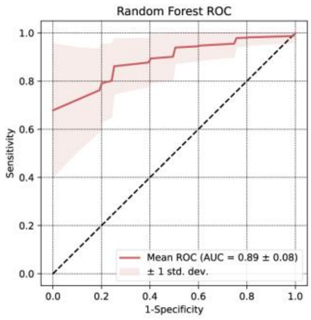

UC2

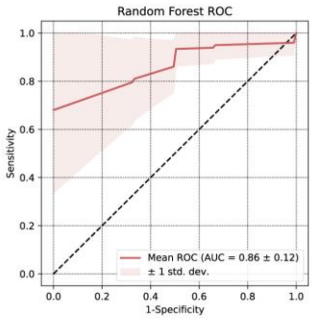

UC3

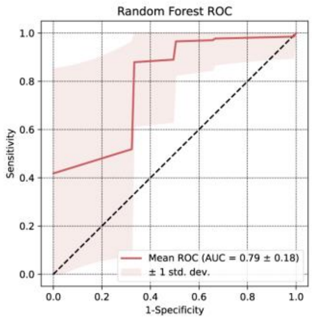

CD1

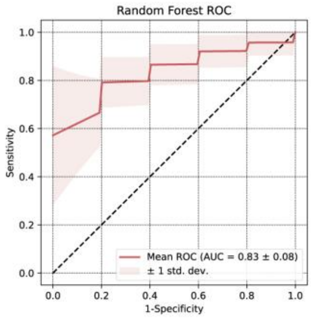

CD2

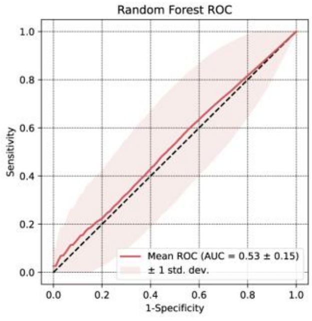

AD

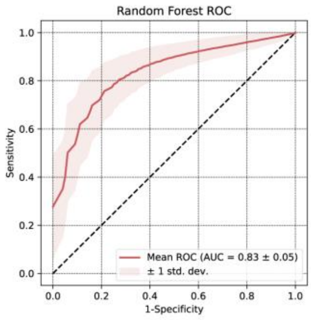

PD

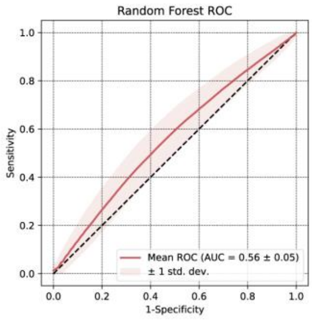

Minor Comments
Line 33 hasn’t: please change to has not
Line 55 Crohn’s diseases (CD) and Alzheimer’s diseases (AD): please change to disease
Lines 58- 60 Phrasing needs revising - “Correlation between diseases” is too vague. Which diseases? Correlation of what between them?
Lines 79 -81 “networks encode richer information than the isolated single layer counterparts, multiplex networks are special cases of multi-layer networks where individual nodes are only connected to themselves across layers”: please can you rephrase
Lines 90-94 “We initially constructed the multi-disease gene co-expression networks in the form of multiplex networks with one layer representing gene co-expression of periodontitis and another one for one of the investigated chronic inflammatory diseases, and then optimal percolation algorithms were applied to obtain the parsimonious omic biomarker set for each constructed multi-disease gene co-expression network”: please can you rephrase
Line 119 largest: please change to larger
Line 157 In line with previous, PMGCN genes may serve as a: please delete as
Line 207 periodontists: please change it
Line 214 periodontists: please change it
Line 222 Crohn’s diseases: please change to disease
Lines 286-287 “addition, PTGR1 also presents complementarity with ComDEGs and DEGs, and the samples tend to lie diagonally in the corresponding scatterplots”: please can you rephrase
Lines 384-385 “these genes might present orthogonal functionality and together contribute to improve power for disease prediction”: please can you rephrase

Lines 393-394 “Notably, our study also confirmed that CXCL5 expressed in fibroblasts displays a vital role in connecting with periodontitis and UC”: please can you rephrase
Line 586 “python”: please change to Python
Lines 606-607 “and then only the links, the nodes of whom belong to the same component in all the layers, are reserved”: please can you rephrase
Line 613 “formular”: please change to formula
Lines 636-637 “The effects of performance on the prediction of chronic inflammatory diseases were accessed based on the Random Forest classifications”: please can you rephrase
Lines 643-647 “model were calculated, boxplots were generated for visualizations”: please can you rephrase
Line 646 “performances”: please can you change to ‘performance metrics’
Line 646 “(AUROC, F1-Score, Accuracy)”: please can you change to ‘and

Accuracy'
Line 651 "performance" : please can you change to 'performance metrics'
Line 652 "Accuracy" : please can you change to 'and Accuracy'

Response:
We thank the reviewer for their meticulous attention to detail. All minor comments regarding grammar, spelling, and phrasing have been addressed in the revised manuscript.

We believe that our detailed responses and the subsequent revisions to the manuscript have significantly improved its clarity, robustness, and overall impact. We are grateful for the opportunity to resubmit our work.

Reviewer #3 (Remarks to the Author):

I co-reviewed the paper with one of the reviewers, as part of the ECR initiative.

Reference:

1. Liu, B. *et al.* An entropy-based metric for assessing the purity of single cell populations. *Nat. Commun.* **11**, 3155 (2020).
2. Kunath, B. J., De Rudder, C., Laczny, C. C., Letellier, E. & Wilmes, P. The oral–gut microbiome axis in health and disease. *Nat. Rev. Microbiol.* **22**, 791–805 (2024).
3. Lam, G. A. *et al.* The oral-gut axis: periodontal diseases and gastrointestinal disorders. *Inflamm. Bowel Dis.* **29**, 1153–1164 (2023).
4. Yang, Y. *et al.* Pan-cancer single-cell dissection reveals phenotypically distinct B cell subtypes. *Cell* **187**, 4790-4811. e22 (2024).

Reviewer #1's Comment 1:
"Overall, the manuscript has been largely improved after addressing all of our questions. This manuscript is more likely to be a biological analysis scope instead of a generalized method since in terms of the network construction goal, this has been attempted by many generic network methods such as the one mentioned WGCNA and CellNet that are developed and benchmarked in more contexts."

Response:
We thank the reviewer for acknowledging the improvements in our manuscript. We agree with the reviewer's assessment that the manuscript is best positioned as a biological, disease-centric network analysis pipeline**.** We have refined the Introduction and Discussion sections to clarify the scope of our contribution. Specifically, we emphasize that our work presents a tailored analytical pipeline for mining biomarkers in chronic inflammatory diseases based on their associations with periodontitis, rather than a general-purpose network construction and analysis tool.

Reviewer #1's Comment 2:
"As a methodology, the software is not well-maintained as a ready-to-go package and is not comprehensively benchmarked under more association of diseases,... I again strongly recommended uploading the data to somewhere open-source platform and make the codebase more distributable and installable."

Response:
We acknowledge this concern and appreciate this practical recommendation. The current implementation was developed as a research-oriented analytical pipeline rather than a fully mature software package. Nevertheless, to improve transparency, reproducibility, and accessibility, we have prepared the data and code for public release.
Actions taken:
All analysis scripts and workflows have been organized into a public repository (**GitHub**: https://github.com/felixshiyong/PMGCN). We have cleaned and restructured the codebase to make it more modular and installable. Readme file, installation instructions and dependency requirements have been added. To enhance the reproducibility of the code, a permanent archival version of the processed expression matrices for network generation have been deposited in an open-access repository (**Zenodo DOI:** 10.5281/zenodo.18953072).

Reviewer #1's Comment 3:
"I wonder how many network methods are really addressing this unique angle"

Response:
We agree that individual elements of our PMGCN framework (e.g., network construction or multiplex network representations) have been explored in prior studies across diverse domains. However, the **integration of multiplex network representation with percolation analysis to specifically identify new biomarkers for chronic inflammatory diseases** is, to our knowledge, rarely addressed. We have **added statement in the Discussion** that clarifies the novelty: it is the *integration* (multiplex network representation + percolation transition analysis) that yields new biological insights.

Reviewer #1's Comment 4:
"On the other hand, the uniqueness of this study is addressing inflammatory disease association with periodontitis in a conceptually different ways: percolation with multi-layer networks representation. Authors may need to highlight their efforts in this angle of biological discoveries instead of comparing to the generic network method, e.g. how robustness is their discoveries of the three markers and their association with the percolation, this may need some validation."

Response:
We appreciate the reviewer's suggestion and have strengthened our manuscript by adding further robustness analyses to support the three candidate markers and their relationship with percolation behavior.

Parameter Sensitivity:
We evaluated the percolation process under different thresholds for network construction (e.g., varying probability cutoffs) while maintaining a reasonably sized network. Under these varying conditions, two of the three genes were consistently identified as critical nodes during the percolation process, demonstrating the robustness of the identified biomarkers. These results have been included in **Supplementary Data File 2**.

Notably, none of the three markers appeared in the set of commonly differentially expressed genes for Periodontitis and UC, despite their consistent identification via percolation analysis. This supports the specific association of these biomarkers with percolation dynamics.

We have revised the Results and Discussion sections accordingly to emphasize this aspect and to reduce direct comparisons with generic network methods.

Reviewer #1's Comment 5:
"They also need to highlight the periodontitis association in the title."

Response:
We have revised the manuscript title to explicitly highlight the periodontitis association.
Revised title:
"Identifying omic biomarkers for chronic inflammatory diseases associated with periodontitis using Percolation on Multi-disease Gene Co-expression Networks"

Editor's Comment 1:
1. Although your study uses database information, it is still considered human research. We require a statement on ethical approval and consent be added to a subsection of the Methods (ex. Ethics Approval or Ethical Consideration as subsection title). If no ethical approval was sought please state something similar to the following. "No ethical approval was sought/required for this study as the genetic information accessed/used was de-identified and publicly available."

Response:

Thank you for the comment. We have added a new subsection titled "**Ethical Consideration**" in the Methods section, which includes the following statement:
"**No ethical approval was sought/required for this study as the genetic information accessed/used was de-identified and publicly available.**"

Editor's Comment 2:
2. Please add the github link in the "Code Availability Statement" to your main reference list and cite where mentioned in this statement.

Response:
Thank you for pointing this out. We have included the GitHub link as a full citation in the main reference list. The corresponding citation has been cited where mentioned.

We believe these revisions address all concerns raised by the reviewer and editors and improve the clarity, rigor, and positioning of the manuscript. We sincerely thank the reviewer and the editors for their insightful comments.
